# Isolation, Structural Elucidation, and Anti‐Melanogenic Activity of a Novel Pectic Polysaccharide From *Alhagi camelorum* Fisch

**DOI:** 10.1002/cbdv.71878

**Published:** 2026-10-30

**Authors:** Zhitao Liu, Shuyan Han, Nannan Xu, Nuermaimaiti Abudukelimu, Jiangyu Zhao

**Affiliations:** ^1^ State Key Laboratory Basis of Xinjiang Indigenous Medicinal Plants Resource Utilization Xinjiang Technical Institute of Physics and Chemistry, Chinese Academy of Sciences Urumqi China; ^2^ University of Chinese Academy of Sciences Beijing China; ^3^ Qinghai Normal University Xining China

**Keywords:** *Alhagi camelorum* Fisch, anti‐melanogenesis, pectic polysaccharide, structural characterization

## Abstract

Hyperpigmentation disorders motivate the search for structurally defined and effective natural depigmenting agents from plant‐derived polysaccharides. The present study characterized the structural features and evaluated the anti‐melanogenic activity of ACP‐II‐1, a novel pectic polysaccharide isolated from *Alhagi camelorum* Fisch. ACP‐II‐1 had a molecular weight of 26.145 kDa and contained GalA, Rha, Gal, Ara, GlcA, and Xyl at respective molar proportions of 0.798, 0.072, 0.053, 0.052, 0.016, and 0.009. Integrated monosaccharide, methylation, FT–IR, and NMR analyses indicated a GalA‐rich structure dominated by homogalacturonan (HG)‐type →4)‐α‐D‐Gal*p*A‐(1→ sequences, with less abundant branched rhamnogalacturonan‐I (RG‐I)‐type motifs and heterogeneous neutral side‐chain motifs. In forskolin‐stimulated B16F10 cells, ACP‐II‐1 significantly lowered melanin content and cellular tyrosinase activity; it also decreased pigmentation and tyrosinase activity in zebrafish. These effects in B16F10 cells coincided with lower MITF, TYR, TRP‐1, and TRP‐2 protein levels, together with altered readouts associated with GSK‐3β/β‐catenin, PKA/CREB, and p38/JNK signaling. Collectively, these findings support the anti‐melanogenic potential of ACP‐II‐1 and warrant further validation in mammalian and human‐relevant skin models, together with comprehensive safety evaluation.

## Graphical abstract

Polysaccharide extraction from *Alhagi camelorum* Fisch. was optimized, yielding ACP‐II‐1, a novel GalA‐rich, HG‐dominant pectin (26.145 kDa) containing branched RG‐I domains. ACP‐II‐1 reduced melanogenesis in forskolin‐stimulated B16F10 cells and a zebrafish model. These effects were accompanied by lower MITF, TYR, TRP‐1, and TRP‐2 protein levels and changes in readouts associated with GSK‐3β/β‐catenin, PKA/CREB, and p38/JNK signaling.

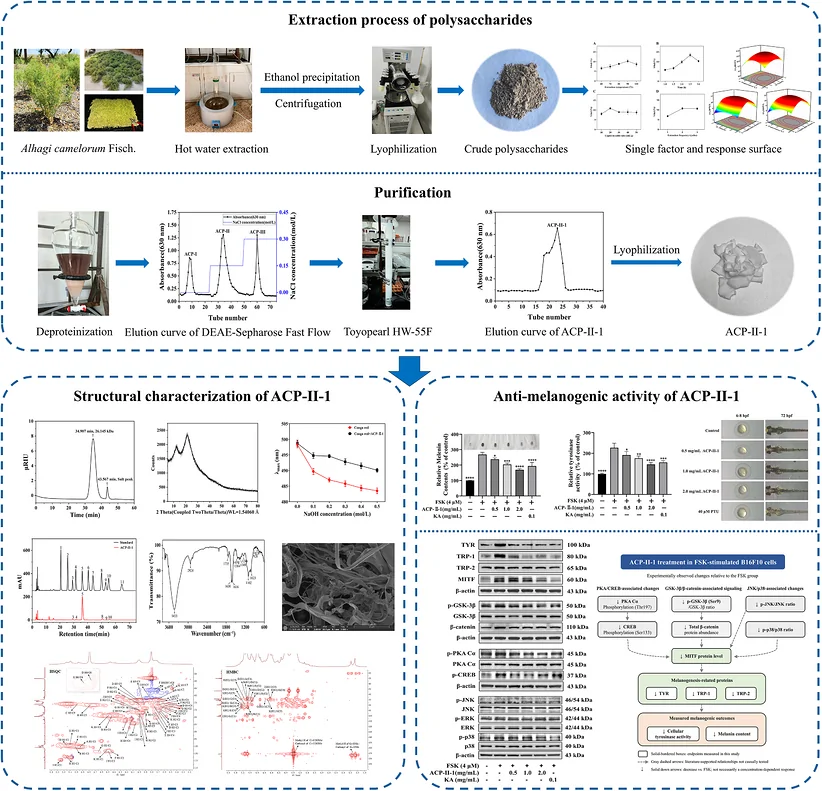

## 1 Introduction

Melanin production and distribution between epidermal melanocytes and keratinocytes are among the principal determinants of human skin pigmentation [1]. The core process of melanogenesis begins in melanosomes with L‐tyrosine as the foundational substrate. As the rate‐limiting enzyme, tyrosinase (TYR) catalyzes this critical step with assistance from TYR‐related proteins 1 and 2 (TRP‐1, TRP‐2) [2]. MITF (microphthalmia‐associated transcription factor) acts as the master regulator by directly activating these key melanogenic enzymes and thereby coordinating the overall melanin biosynthesis program [3]. Under physiological conditions, neighboring keratinocytes receive the synthesized melanin, which is eventually degraded during epidermal turnover [4]. However, its overproduction is a direct cause of hyperpigmentation disorders [1]. Owing to substantial market demand for skin‐whitening agents, TYR‐targeting compounds, including arbutin and kojic acid (KA), have become mainstream ingredients. However, growing clinical evidence of adverse effects associated with their prolonged use has intensified the pursuit of safer, natural alternatives [5, 6].

Polysaccharides of plant origin have garnered considerable interest as prospective therapeutic agents because of their favorable biocompatibility, limited toxicity, and diverse bioactivities such as antioxidant, inflammation‐modulating, and anti‐TYR properties [7, 8, 9]. Evidence indicates that several polysaccharides can attenuate melanogenesis through mechanisms beyond direct TYR inhibition, particularly by regulating upstream pathways involved in MITF expression, including the MAPK, Wnt/β‐catenin, and cAMP cascades [10, 11]. This multi‐targeted mechanism presents a significant therapeutic advantage.

*Alhagi camelorum* Fisch. is the sole Chinese species of the *Alhagi* genus and is a perennial Fabaceae subshrub known as camel thorn [12]. Morphologically, the plant has erect, multi‐branched stems, rigid leaves, sharp thorn‐like inflorescence axes, and a well‐developed root system; it also exhibits remarkable tolerance to drought, salinity, and nutrient‐poor soils. Within China, its distribution is concentrated in Xinjiang, Gansu, Inner Mongolia, and Qinghai [12]. Valued for its role in forage production and desert ecosystem stabilization, it also holds a place in traditional medicine [13, 14]. Polysaccharides are considered its primary bioactive constituents, with studies confirming their notable anti‐inflammatory and antioxidant activities [15, 16]. Because inflammation and oxidative stress are both closely linked to melanogenesis induction [17, 18], we hypothesize that *A. camelorum* polysaccharides may possess significant anti‐melanogenic efficacy. Nevertheless, this potential activity and its underlying structure‐activity relationship (SAR) remain unexplored.

Therefore, the present work involved the isolation of the predominant polysaccharide fraction from *A. camelorum*, followed by structural elucidation and assessment of its anti‐melanogenic activity. A response surface methodology (RSM) design was employed to determine suitable extraction parameters, and the crude polysaccharide obtained was termed ACP (*A. camelorum* polysaccharide). The major fraction obtained by anion‐exchange chromatography was designated ACP‐II and subsequently purified by size‐exclusion chromatography, yielding the first purified subfraction, ACP‐II‐1. Its structure was characterized through complementary compositional, spectroscopic, and linkage analyses. Anti‐melanogenic activity was evaluated in forskolin (FSK)‐stimulated B16F10 cells and zebrafish, while changes in melanogenesis‐related proteins and signaling‐associated readouts were examined in the cellular model. By integrating structural characterization with biological evaluation in B16F10 cells and zebrafish, we sought to assess the anti‐melanogenic activity of ACP‐II‐1 and provide a preliminary basis for its further evaluation as a natural supplement for hyperpigmentation management.

## 2 Results and Discussion

### 2.1 Optimization of Polysaccharide Extraction

#### 2.1.1 Single Factor Test

Single‐factor tests were conducted to determine preliminary ranges for temperature, time, liquid‐to‐solid ratio, and cycle number (Figure S1). The ACP yield initially increased with rising temperature and prolonged time, peaking at 90°C and 2.5 h before declining, indicating potential thermal degradation and polysaccharide compromise under extreme conditions [19, 20]. In contrast, the liquid‐to‐solid ratio showed a distinct optimal point at 20 mL/g, while the yield reached a maximum after two extraction cycles, demonstrating diminishing returns thereafter.

#### 2.1.2 RSM Extraction

A Box–Behnken design was applied to identify suitable process parameters (Tables S1 and S2). The observed responses were adequately described by a statistically significant quadratic model (*p* = 0.0015); the lack‐of‐fit test was nonsignificant (*p* = 0.9606), indicating adequate model fit (Table S3). Its high *R* ^2^ (0.9420) and low coefficient of variation (4.27%) further attested to the model's reliability. The derived second‐order polynomial equation was:

$$\begin{array}{ccc} Yield & = & -80.76+1.413\times A+12.19\times B+1.134\times C-0.019\\ & & \;\times AB-0.004\times AC-0.031\times BC-0.007\times A^{2}-1.950\\ & & \;\times B^{2}-0.017\times C^{2} \end{array}$$

Here, A, B, and C represent temperature (°C), time (h), and the liquid‐to‐solid ratio (mL/g), respectively. ANOVA showed that, among the quadratic components, C^2^ (the liquid‐to‐solid ratio) had the largest effect on yield (*p* < 0.0001). The A^2^ (temperature, *p* = 0.0090) and B^2^ (time, *p* = 0.0429) terms ranked next, whereas no interaction was significant (all *p* > 0.05). Inspection of the three‐dimensional response surfaces (Figure S2) was consistent with the fitted model and identified an optimum combination of 91.17°C, 2.52 h, and a ratio of 20.90 mL/g, corresponding to a predicted maximum yield of 10.88%. Validation experiments under these optimized conditions (*n* = 3) yielded an actual yield of 10.72% ± 0.31%, confirming the model's accuracy.

### 2.2 Isolation, Purification and General Characterization of ACP‐II‐1

Following protein removal and dialysis, ACP was fractionated using a DEAE‐Sepharose Fast Flow column, producing three fractions, ACP‐I, ACP‐II, and ACP‐III (Figure 1A). Their yields and anti‐melanogenic activities at 1.0 mg/mL were compared (Table S4); ACP‐II showed the highest yield (38.77%) and strongest melanin inhibition (28.72% ± 2.37%), whereas ACP‐I showed a slight stimulatory effect (−5.37% ± 1.49%) and ACP‐III exhibited modest activity (9.07% ± 1.21%). Accordingly, ACP‐II was selected for further purification by size‐exclusion chromatography on a Toyopearl HW‐55F column, and the first recovered subfraction was designated ACP‐II‐1 (Figure 1B). Subsequent high‐performance gel‐permeation chromatography (HPGPC) yielded one symmetric peak (Figure 1C), supporting the homogeneity of ACP‐II‐1. Using the dextran calibration relationship shown in Figure S3A, the *M*w of ACP‐II‐1 was calculated as 26.145 kDa, with an *M*w/*M*n ratio of 1.59. No detectable signal was observed at either 260 or 280 nm in the UV scan (Figure S3B), suggesting negligible contamination by nucleic acids or proteins and supporting the purity of ACP‐II‐1.

**FIGURE 1:**
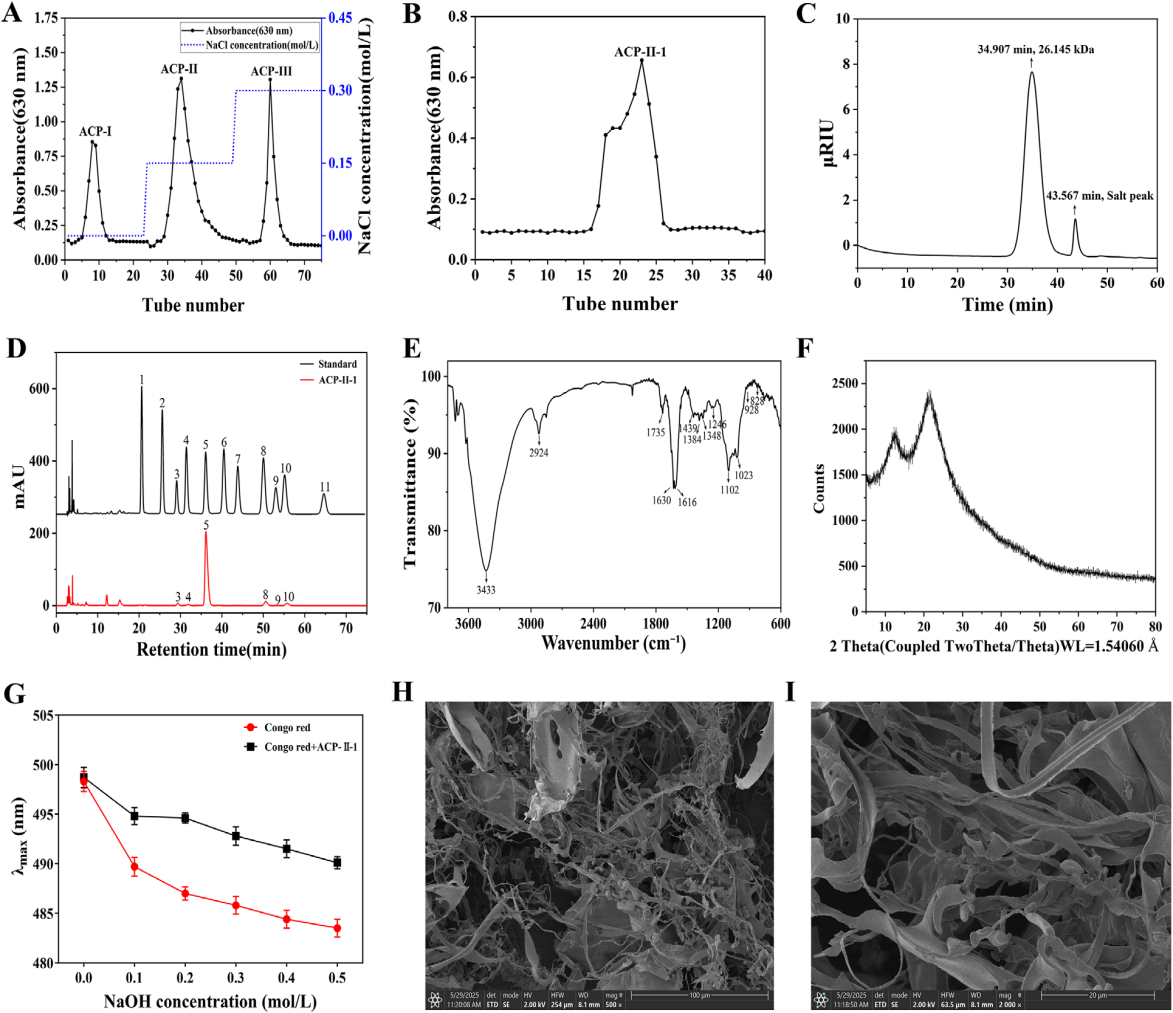
Separation and physicochemical profiling of ACP‐II‐1. (A) Step‐gradient elution trace for ACP during fractionation on a DEAE‐Sepharose Fast Flow anion‐exchange column. (B) Elution trace for ACP‐II during purification on a Toyopearl HW‐55F size‐exclusion column. (C) HPGPC profile of ACP‐II‐1. (D) HPLC profiles of the PMP precolumn derivatives prepared from ACP‐II‐1 and monosaccharide standards (peak assignments 1–11: Man, GlcN, Rha, GlcA, GalA, GalN, Glc, Gal, Xyl, Ara, and Fuc, respectively). (E) FT‐IR spectrum of ACP‐II‐1. (F) XRD pattern of ACP‐II‐1. (G) Congo red assay of ACP‐II‐1. (H, I) SEM micrographs of ACP‐II‐1 acquired at 500× and 2000× magnification, respectively.

### 2.3 Monosaccharides Composition of ACP‐II‐1

Six monosaccharides were detected in ACP‐II‐1: GalA, Rha, Gal, Ara, GlcA, and Xyl. Their respective molar proportions were 0.798, 0.072, 0.053, 0.052, 0.016, and 0.009 (Figure 1D). This remarkably high GalA content (79.8%) distinctly sets ACP‐II‐1 apart from other reported acidic polysaccharides in camel thorn species. For instance, Chen et al. and Manafu et al. described polysaccharides from *A. camelorum* with GalA contents of only 0.59% and 54.92%, respectively, and a much more diverse monosaccharide profile [21, 22]. Similarly, a polysaccharide from *Alhagi pseudalhagi* exhibited a balanced composition with GalA representing only a minor fraction [23]. These pronounced discrepancies strongly suggest that ACP‐II‐1 is a pectic polysaccharide, potentially rich in homogalacturonan (HG). The observed variations likely originate from intrinsic factors such as plant species and organ specificity, as well as extrinsic factors including extraction and purification methodologies.

### 2.4 Structure Elucidation of ACP‐II‐1

#### 2.4.1 FT–IR Analysis

ACP‐II‐1 exhibited the characteristic FT–IR features of polysaccharides (Figure 1E). The broad feature at 3433 cm^−1^ was attributed to O─H stretching [9], whereas the 2924 cm^−1^ feature reflected C─H stretching [24]. The 1735 and 1616 cm^−1^ signals were assigned to methyl‐esterified carbonyl (COO─CH_3_) and carboxylate (COO^−^) stretching, respectively, consistent with the presence of uronic acid residues [25]. Other bands were associated with bound water (1630 cm^−1^) [26], C─H deformation (1439 cm^−1^) [27], symmetric C═O stretching (1384 and 1348 cm^−1^) [19], and weak uronic‐acid‐ or sulfate‐ester‐related vibrations (1246 cm^−1^) [28]. Absorptions at 1102 and 1023 cm^−1^ were indicative of pyranose‐ring vibrations [29]. Weak bands at 928 and 828 cm^−1^ provided evidence for β‐ and α‐configurations, respectively, suggesting that both glycosidic configurations may occur in ACP‐II‐1 [30]. Overall, the FT–IR profile was consistent with an acidic, pyranose‐rich polysaccharide containing both α‐ and β‐linked residues. These FT–IR assignments were consistently corroborated by subsequent NMR analysis: the ^13^C NMR signals at δ 175.2 and 171.5 ppm confirmed the presence of uronic acids, the anomeric proton signals distributed on both sides of δ 4.9 ppm verified the coexistence of both glycosidic configurations, and HSQC correlations provided unambiguous assignment of pyranose ring carbons. The complementary nature of FT–IR and NMR thus provides a robust structural foundation for ACP‐II‐1.

#### 2.4.2 Methylation Analysis

PMAA–GC–MS analysis (Table 1 and Figure S3G) revealed the linkage patterns of ACP‐II‐1. The principal backbone linkage was 1,4‐linked Gal*p*A (74.42%), indicative of a HG domain [31]. The presence of 1,2‐linked Rha*p* (3.42%) and 1,2,4‐linked Rha*p* (3.83%), totaling 7.25%, confirmed the existence of rhamnogalacturonan‐I (RG‐I) domains, where the 1,2,4‐linked Rha*p* serves as a branching point [32, 33]. The side chains were compositionally diverse: Arabinan segments were suggested by terminal (t)‐Ara*f* (2.19%) and 1,5‐linked Ara*f* (3.03%). Galactan side chains were evidenced by t‐Gal*p* (2.67%) and 1,3,6‐linked Gal*p* (2.55%) residues. The detection of 1,4‐linked Xyl*p* (1.22%) indicated the presence of short xylan segments, and terminal Glc*p*A (1.20%) likely capped some side chains. Collectively, these results confirm that ACP‐II‐1 possesses an HG‐dominant pectic architecture containing branched RG‐I domains decorated with neutral sugar side chains.

**TABLE 1:** Methylation analysis of ACP‐II‐1.

Types of glycosidic linkage	Mw (Da)	RT (min)	PMAA	Relative molar ratio (%)
t‐Gal*p*A	325	17.669	1,5‐di‐*O‐*acetyl‐2,3,4,6‐tetra‐*O*‐methyl galactitol	1.79
1,4‐Gal*p*A	353	20.064	1,4,5‐tri‐*O*‐acetyl‐2,3,6‐tri‐*O*‐methyl glucitol	74.42
1,4,6‐Gal*p*A	381	23.761	1,4,5,6‐tetra‐*O*‐acetyl‐2,3‐di‐*O*‐methyl galactitol	3.68
1,2‐Rha*p*	321	15.686	1,2,5‐tri‐*O*‐acetyl‐6‐deoxy‐3,4‐di‐*O*‐methyl rhamnitol	3.42
1,2,4‐Rha*p*	349	18.594	1,2,4,5‐tetra‐*O*‐acetyl‐6‐deoxy‐3‐*O*‐methyl rhamnitol	3.83
t‐Gal*p*	325	17.669	1,5‐di‐*O*‐acetyl‐2,3,4,6‐tetra‐*O*‐methyl galactitol	2.67
1,3,6‐Gal*p*	379	24.606	1,3,5,6‐tetra‐*O*‐acetyl‐2,4‐di‐*O*‐methyl galactitol	2.55
t‐Ara*f*	279	11.851	1,4‐di‐*O*‐acetyl‐2,3,5‐tri‐*O*‐methyl arabinitol	2.19
1,5‐Ara*f*	307	15.623	1,4,5‐tri‐*O*‐acetyl‐2,3‐di‐*O*‐methyl arabinitol	3.03
t‐Glc*p*A	325	16.962	1,5‐di‐*O*‐acetyl‐2,3,4,6‐tetra‐*O*‐methyl glucitol	1.20
1,4‐Xyl*p*	307	15.817	1,4,5‐tri‐*O*‐acetyl‐2,3‐di‐*O*‐methyl xylitol	1.22

*Note*: RT denotes retention time; PMAA refer to partially methylated alditol acetates. Relative molar ratios were obtained by integrating the corresponding chromatographic signals.

#### 2.4.3 NMR Spectroscopy Analysis

The fine structure of ACP‐II‐1 was elucidated through an integrated 1D and 2D NMR analysis. The ^1^H NMR spectrum (Figure 2A) exhibited characteristic anomeric proton signals dispersed on both sides of δH 4.9 ppm, indicating both α‐ and β‐configured glycosidic linkages [29]. The ^13^C NMR spectrum (Figure 2B) showed anomeric carbon signals in δC 95–110 ppm, with a cluster at δC 106–110 ppm characteristic of arabinofuranose (Ara*f*) residues [33]. Two distinct signals at δC 175.2 and 171.5 ppm were assigned to the carbonyl carbons of uronic acids in different esterification environments. Together with the monosaccharide composition results, these signals were consistent with the presence of Gal*p*A and Glc*p*A residues [34].

**FIGURE 2:**
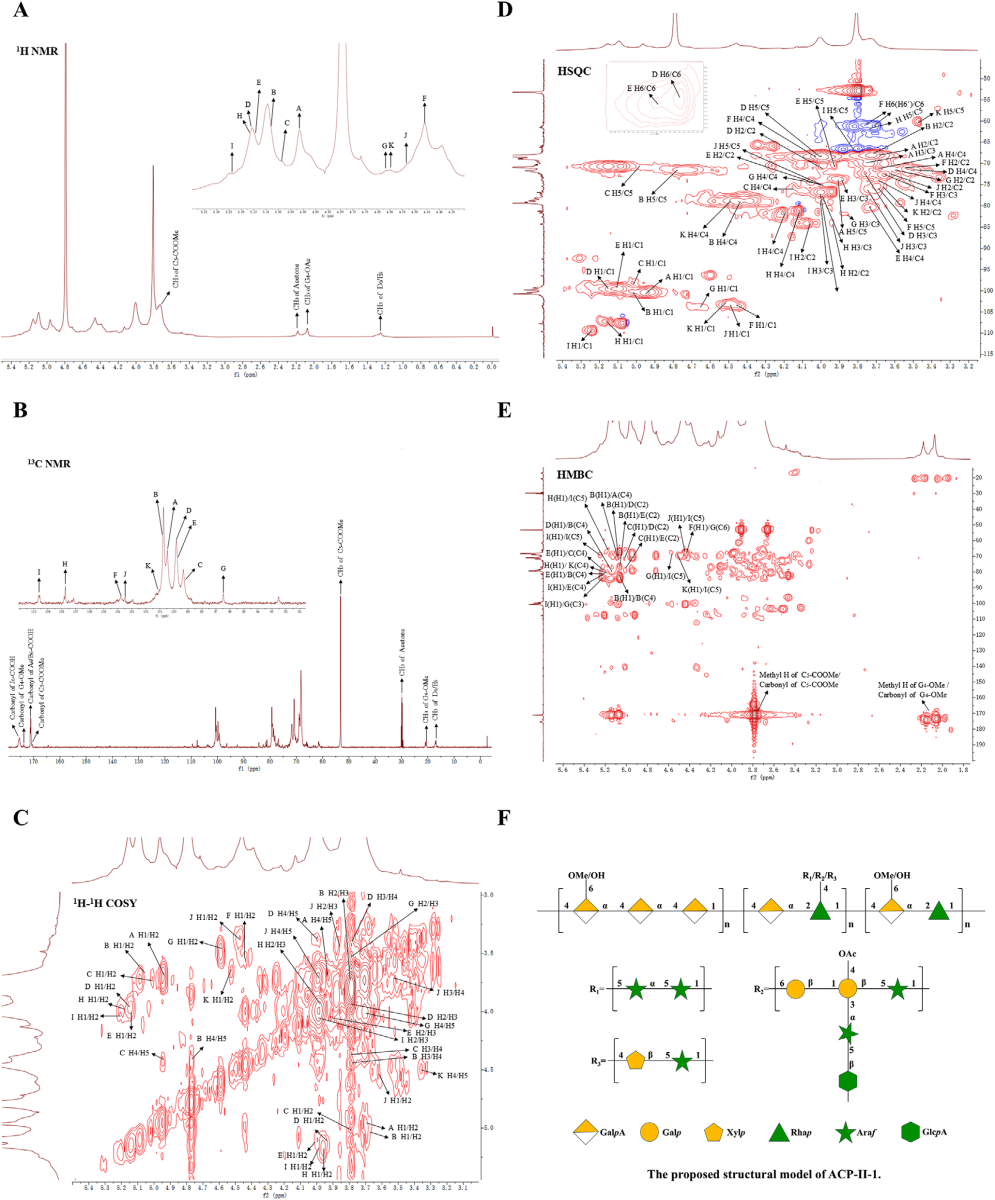
NMR characterization and proposed structural model of ACP‐II‐1. Panels A–E show the ^1^H NMR, ^13^C NMR, ^1^H–^1^H COSY, ^1^H–^13^C HSQC, and ^1^H–^13^C HMBC spectra, respectively; panel F shows the proposed structural model. Residue labels A–K correspond to the assignments in Table 2. R_1_–R_3_ indicate representative side‐chain motifs; OMe and OAc denote methyl ester and O‐acetyl substituents, respectively.

Key structural features were identified through spectral‐editing and correlation NMR experiments. The DEPT‐135 spectrum (Figure S3E) revealed unsubstituted primary hydroxymethyl groups (─CH_2_OH) at δC 62.15, 61.22, and 61.27 ppm [24]. HSQC correlations at δH/δC 1.23/16.64 and 1.28/16.85 ppm (Figure 2D) confirmed the presence of Rha*p* methyl groups. Complete signal assignment (Table 2) was achieved via COSY (Figure 2C), TOCSY (Figure S3F), and HSQC analysis.

**TABLE 2:** Resonance assignments based on ^1^H and ^13^C NMR data for the glycosyl residues of ACP‐II‐1.

Code	Glycosyl residues	Chemical shift, *δ* (ppm)							
		H1/C1	H2/C2	H3/C3	H4/C4	H5(H5')/C5	H6(H6')/C6	OMe/O‐CH3	OAc/CH3CO CH3CO
A	α‐D‐Gal*p*A‐(1→	4.96	3.7	3.77	3.69	3.93			
		100.48	68.46	70.48	71.42	73.74	170.98		
B	→4)‐α‐D‐Gal*p*A‐(1→	5.05	3.68	3.79	4.43	4.77			
		100.13	67.32	69.12	81.67	71.75	171.13		
C	→4)‐α‐D‐Gal*p*A‐6‐OMe‐(1→	5.01	3.73	3.78	4.36	4.96		3.74	
		99.51	68.35	68.57	78.87	70.93	171.09	53.19	
D	→2)‐α‐L‐Rha*p*‐(1→	5.15	3.96	3.78	3.4	4	1.23		
		99.78	70.71	72.58	71.04	70.25	16.64		
E	→2,4)‐α‐L‐Rha*p*‐(1→	5.13	4.01	3.91	3.71	3.93	1.28		
		99.77	76.58	73.89	80.49	70.87	16.85		
F	β‐D‐Gal*p*‐(1→	4.44	3.54	3.65	3.99	3.69	3.71(3.77)		
		103.54	71.64	72.77	70.68	75.23	61.22		
G	→3,6)‐4‐OAc‐β‐D‐Gal*p*‐(1→	4.60	3.52	3.85	5.15	3.78	3.80(3.64)		2.07/20.54
		103.83	70.24	81.71	73.18	75.21	66.39		173.74
H	α‐L‐Ara*f*‐(1→	5.18	3.95	3.99	4.1	3.72(3.86)			
		107.62	83.96	79.09	81.15	62.15			
I	→5)‐α‐L‐Ara*f*‐(1→	5.22	4.05	3.99	4.18	3.80(3.88)			
		109.23	84.18	79.12	82.05	66.89			
J	β‐D‐Glc*p*A‐(1→	4.49	3.43	3.78	3.71	3.99			
		103.35	71.75	75.09	73.88	74.97	175.28		
K	→4)‐β‐D‐Xyl*p*‐(1→	4.52	3.65	3.78	4.47	3.45			
		101.01	75.46	72.95	79.58	61.27			

Gal*p*A was the predominant residue. The H1/C1 signals of α‐D‐Gal*p*A residues (A, B, C) were assigned to δH/δC 4.96/100.48, 5.05/100.13, and 5.01/99.51 ppm, respectively [24, 35, 36]. The characteristic downfield shift of H4/C4 in 1,4‐linked Gal*p*A residues (B and C, δH ∼4.3–4.5, δC 76–82 ppm) versus terminal Gal*p*A (A, δH/δC 3.69/71.42 ppm) confirmed glycosylation at C‐4. Methyl esterification at C‐6 of residue C was evidenced by signals at δH/δC 3.74/53.19 (─OCH_3_) and δC 171.09 ppm (C═O) [37]. O‐Acetylation at C‐4 of residue G was confirmed by signals at δH/δC 2.07/20.54 and 2.07/173.44 ppm [38]; its deshielded C‐3 (δC 81.71 ppm) indicated glycosylation.

The HMBC spectrum (Figure 2E) showed key inter‐residue correlations supporting the backbone linkage assignments. A correlation from the anomeric proton of one 1,4‐linked α‐D‐Gal*p*A residue (B H1, δH 5.05) to the C‐4 of a preceding Gal*p*A residue (δC 81.67 ppm) confirmed the →4)‐α‐D‐Gal*p*A‐(1→4)‐α‐D‐Gal*p*A linkage characteristic of the HG region [31]. The alternating RG‐I sequence was proven by two reciprocal correlations between B H1 and D C2 (δH 5.05/δC 70.71 ppm), and between D H1 and B C4 (δH 5.15/δC 81.67 ppm). This confirms the repeating →4)‐α‐D‐Gal*p*A‐(1→2)‐α‐L‐Rha*p*‐(1→ disaccharide unit of the RG‐I backbone [29, 39]. In addition, the correlations from I H1 (δH 5.22) to C‐4 of →2,4)‐α‐L‐Rha*p* (δC 80.49 ppm), together with the methylation results, supported attachment of an Ara*f*‐containing side chain at O‐4 of this Rha*p* residue.

Additional HMBC correlations indicated several local side‐chain motifs. Together with the linkage analysis, the I(H1)/I(C5) and H(H1)/I(C5) correlations at δH/δC 5.22/66.89 and 5.18/66.89 ppm, respectively, were consistent with (1→5)‐linked Ara*f* segments containing terminal Ara*f* residues. The H(H1)/K(C4) and K(H1)/I(C5) correlations at δH/δC 5.18/79.58 and 4.52/66.89 ppm supported an Ara*f*‐(1→4)‐Xyl*p*‐(1→5)‐Ara*f* local sequence. The F(H1)/G(C6) correlation at δH/δC 4.44/66.39 ppm indicated a β‐D‐Gal*p*‐(1→6)‐β‐D‐Gal*p* motif. Correlations G(H1)/I(C5), J(H1)/I(C5), and I(H1)/G(C3) at δH/δC 4.60/66.89, 4.49/66.89, and 5.22/81.71 ppm, respectively, further supported local Gal*p*–Ara*f* linkages and a possible terminal Glc*p*A‐(1→5)‐Ara*f* motif. These correlations indicate heterogeneous Ara*f*‐, Xyl*p*‐, Gal*p*‐, and Glc*p*A‐containing side‐chain motifs rather than three uniquely defined side‐chain sequences.

Taken together with the monosaccharide data and methylation findings, the NMR results indicate that ACP‐II‐1 is a GalA‐rich pectic polysaccharide dominated by HG‐type →4)‐α‐D‐Gal*p*A‐(1→ sequences and containing less abundant RG‐I‐type →4)‐α‐D‐Gal*p*A‐(1→2)‐α‐L‐Rha*p*‐(1→ motifs. Some →2,4)‐α‐L‐Rha*p*‐(1→ residues carry heterogeneous Ara*f*‐, Xyl*p*‐, Gal*p*‐, and Glc*p*A‐containing side‐chain motifs at O‐4. Methyl‐esterified Gal*p*A and an O‐acetyl substituent were also detected. The proposed motif‐level structural model is shown in Figure 2F; however, the precise arrangement and complete sequences of these motifs remain unresolved.

#### 2.4.4 SEM‐EDX and XRD Results

SEM imaging revealed the surface ultrastructure of ACP‐II‐1. At 500× magnification, the structure appeared loose with overlapping fine filaments and flakes (Figure 1H). At 2000×, the surface was smooth with hair‐like fibrils and localized spherical structures (Figure 1I). EDX analysis confirmed a composition of 55.0% C and 43.2% O, with less than 2% Na and Ca (Figure S3C), indicating high carbohydrate purity. The XRD pattern of ACP‐II‐1 (Figure 1F) exhibited two characteristic diffraction peaks at approximately 12° and 22° (2θ), typical for semi‐crystalline pectin [40].

#### 2.4.5 Congo Red Analysis

Conventionally, triple‐helical structure is confirmed by a Congo red binding assay, where complex formation induces a redshift in λ_max_ that is disrupted under high alkalinity, causing a blueshift [41]. Intriguingly, the Congo Red‐ACP‐II‐1 complex exhibited a continuous redshift with increasing NaOH concentration (0 to 0.5 mol/L) (Figure 1G), contrasting with conventional behavior and indicating the lack of a triple‐helix structure [42].

### 2.5 Cell Viability and Anti‐Melanogenic Activity of ACP‐II‐1 in FSK‐Stimulated B16F10 Cells

Cell viability in FSK‐stimulated B16F10 cells was assessed using the CCK‐8 assay [10] and remained above 90% at 0.05–4.0 mg/mL relative to the normal control (Figure 3A). The broader concentration range was used to establish tolerability, whereas 0.5, 1.0, and 2.0 mg/mL were selected as a twofold series for subsequent efficacy and protein analyses to assess activity across a concentration series at lower, clearly tolerated concentrations. Thus, the study focused on a lower concentration range rather than the highest non‐cytotoxic concentration tested; anti‐melanogenic efficacy at 4.0 mg/mL was not evaluated.

**FIGURE 3:**
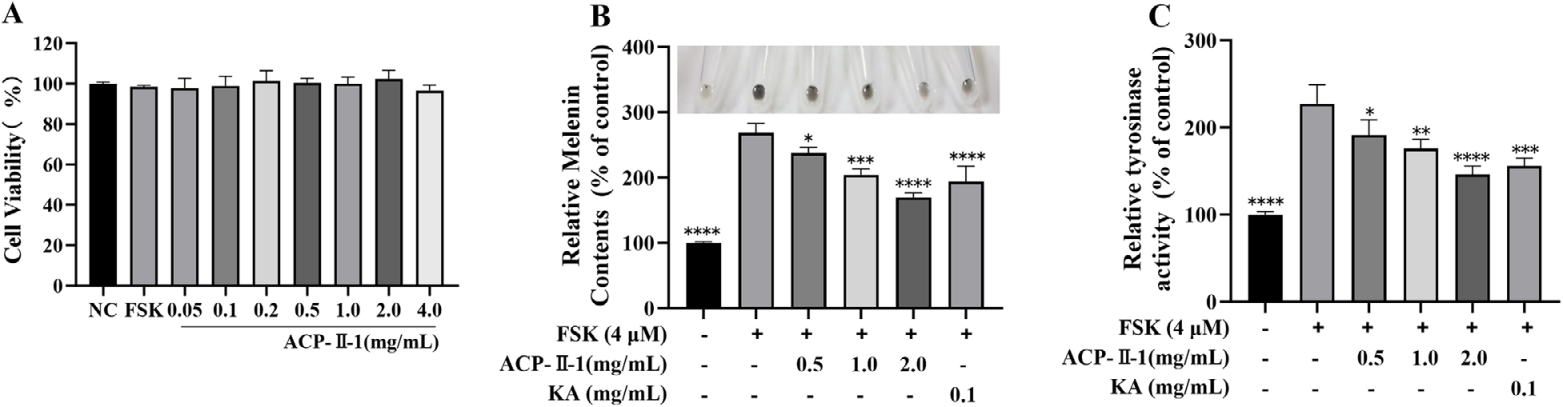
Effects of ACP‐II‐1 on cytotoxicity, melanin accumulation, and cellular tyrosinase activity in FSK‐stimulated B16F10 cells. (A) Viability of FSK‐exposed cells following treatment with different concentrations of ACP‐II‐1. (B) Melanin content and the corresponding visual appearance of cell lysates after treatment for 72 h. (C) Intracellular tyrosinase activity measured after 48 h of treatment. Results are expressed as mean ± SD (*n* = 3). Symbols indicate differences from the FSK group: *p < 0.05, **p < 0.01, ***p < 0.001, and ****p < 0.0001.

To evaluate the anti‐melanogenic activity of ACP‐II‐1, a pigmentation model was established by stimulating B16F10 cells with FSK (4 µM), as described in our previous study [10]. FSK significantly increased melanin content to 268.56% of the normal control level (*p* < 0.0001). ACP‐II‐1 produced concentration‐dependent reductions in melanin content (*p* < 0.01, *p* < 0.001, and *p* < 0.0001 at 0.5, 1.0, and 2.0 mg/mL, respectively), with2 mg/mL resulting in 37.23% inhibition relative to the FSK group. Reduced pigmentation of the cell pellets was consistent with these quantitative results (Figure 3B). ACP‐II‐1 also produced concentration‐related reductions in cellular tyrosinase activity (Figure 3C) by 15.81%, 22.57%, and 35.74% relative to the FSK group at 0.5, 1.0, and 2.0 mg/mL, respectively (*p* < 0.05, *p* < 0.01, and *p* < 0.0001). These concordant changes support an association between lower cellular tyrosinase activity and reduced melanin accumulation.

### 2.6 Effects of ACP‐II‐1 on the Expression of Melanogenesis‐Related Proteins

To investigate the molecular changes associated with the anti‐melanogenic effect of ACP‐II‐1, key melanogenesis‐related proteins were examined by western blot analysis. Consistent with the observed changes in melanin content and tyrosinase activity, FSK elevated MITF and its downstream melanogenic proteins TYR, TRP‐1, and TRP‐2 [10] (Figure 4A–E), whereas ACP‐II‐1 attenuated these increases. MITF, TYR, and TRP‐2 showed concentration‐related decreases. In contrast, TRP‐1 exhibited a non‐monotonic response: its mean level at 2.0 mg/mL was higher than at 1.0 mg/mL, although both remained below that of the FSK group. This pattern may reflect protein‐specific regulation or biological and experimental variability, but its basis cannot be resolved from the single‐time‐point western blot analysis. Thus, ACP‐II‐1 reduced the FSK‐induced increase in TRP‐1 protein abundance without showing a strictly concentration‐dependent response. KA, used as a positive control, also reduced melanogenesis‐associated proteins under the present experimental conditions. Although KA is primarily recognized as a tyrosinase inhibitor, previous studies have shown that KA can also regulate melanogenesis‐related molecules, including MITF and tyrosinase family proteins [43, 44, 45].

**FIGURE 4:**
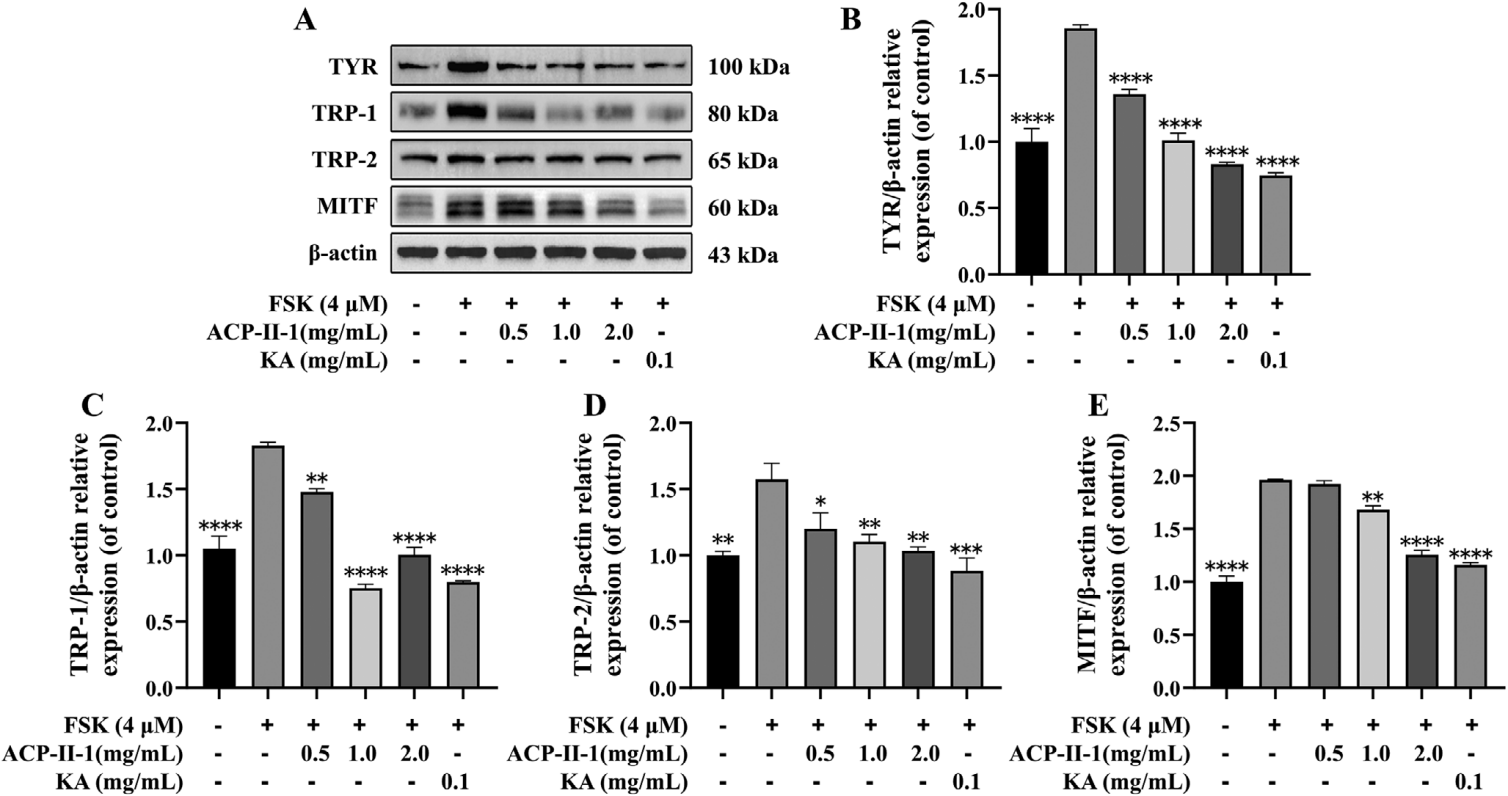
Changes in melanogenesis‐associated proteins after ACP‐II‐1 treatment of FSK‐exposed B16F10 cells. (A) Representative immunoblots of TYR, TRP‐1, TRP‐2, and MITF. (B–E) Densitometric quantification of TYR, TRP‐1, TRP‐2, and MITF, normalized against the control group. Results are expressed as mean ± SD (*n* = 3). Symbols indicate differences from the FSK group: *p < 0.05, **p < 0.01, ***p < 0.001, and ****p < 0.0001.

### 2.7 Effects of ACP‐II‐1 on GSK‐3β/β‐Catenin‐Associated Signaling

GSK‐3β regulates β‐catenin stability, and inhibitory phosphorylation of GSK‐3β at Ser9 can favor β‐catenin accumulation. Stabilized β‐catenin can subsequently promote MITF transcription and melanogenesis [46, 47]. FSK stimulation increased the p‐GSK‐3β (Ser9)/GSK‐3β ratio and β‐catenin abundance (Figure 5A–C). Each tested concentration of ACP‐II‐1 significantly reduced both the p‐GSK‐3β (Ser9)/GSK‐3β ratio and total β‐catenin abundance relative to the FSK group (*p* < 0.05). However, the mean responses at 1.0 and 2.0 mg/mL were similar for both endpoints, suggesting a possible plateau‐like or saturable response rather than a clear concentration‐dependent pattern. This similarity does not demonstrate equivalence, confirm a plateau, or establish that 1.0 mg/mL produces the maximal regulatory effect, particularly because concentrations above 2.0 mg/mL were not examined in the protein analyses. Because these readouts were measured at a single time point and only total β‐catenin was assessed, GSK‐3β enzymatic activity, β‐catenin turnover, and its subcellular localization remain unresolved. Overall, these findings are consistent with modulation of GSK‐3β/β‐catenin‐associated signaling, as previously reported for a *Saussurea involucrata* polysaccharide [10].

**FIGURE 5:**
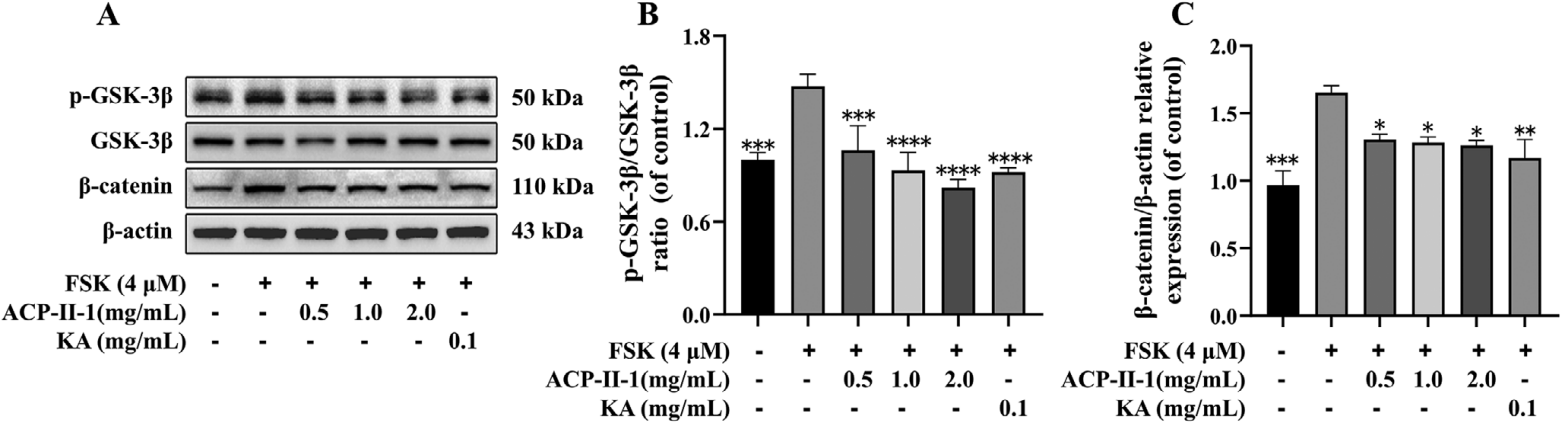
GSK‐3β/β‐catenin responses to ACP‐II‐1 in FSK‐stimulated B16F10 cells. (A) Representative immunoblots of p‐GSK‐3β (Ser9), total GSK‐3β, and β‐catenin. (B–C) Densitometric measurements of the p‐GSK‐3β/GSK‐3β ratio and β‐catenin abundance, expressed relative to the control group. Results are expressed as mean ± SD (*n* = 3). Symbols indicate differences from the FSK group: *p < 0.05, **p < 0.01, ***p < 0.001, and ****p < 0.0001.

### 2.8 Effect of ACP‐II‐1 on PKA/CREB‐Associated Signaling

Activation of adenylyl cyclase by FSK raises intracellular cAMP, which in turn stimulates protein kinase A (PKA). Activated PKA can phosphorylate CREB at Ser133, thereby promoting MITF transcription and melanogenesis [48, 49]. FSK significantly increased the p‐PKA Cα (Thr197)/PKA Cα ratio and the level of p‐CREB (Ser133) (Figure 6A–C), whereas ACP‐II‐1 attenuated both responses in a concentration‐related manner. The parallel reductions in PKA Cα and CREB phosphorylation are consistent with modulation of PKA/CREB‐associated signaling and may contribute to the anti‐melanogenic effects of ACP‐II‐1. Similar phosphorylation changes have been reported for *Saussurea involucrata* polysaccharides [10] and wampee fruit pectin [50].

**FIGURE 6:**
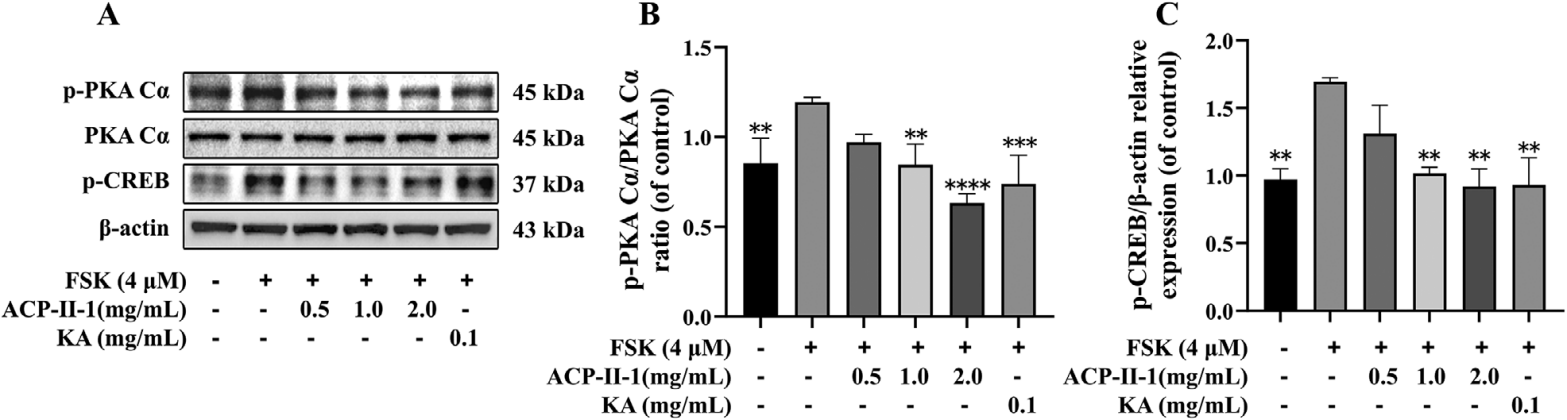
PKA/CREB signaling responses to ACP‐II‐1 in B16F10 cells exposed to FSK. (A) Representative immunoblots of p‐PKA Cα (Thr197), total PKA Cα, and p‐CREB (Ser133). (B, C) Densitometric measurements of the p‐PKA Cα/PKA Cα ratio and p‐CREB abundance, expressed relative to the control group. Results are expressed as mean ± SD (*n* = 3). Symbols indicate differences from the FSK group: **p < 0.01, ***p < 0.001, and ****p < 0.0001.

### 2.9 Effects of ACP‐II‐1 on MAPK‐Associated Signaling

MAPK pathways can differentially regulate melanogenesis: ERK activation can promote MITF degradation [51], whereas p38 and JNK can enhance melanogenic signaling [52, 53]. FSK enhanced JNK and p38 phosphorylation but produced no significant change in ERK phosphorylation or in the total abundance of JNK, p38, and ERK (Figure 7A–D). ACP‐II‐1 decreased the relative phosphorylation of JNK and p38, represented by the p‐JNK/JNK and p‐p38/p38 ratios, respectively, while the p‐ERK/ERK ratio remained statistically unchanged. This pattern is consistent with modulation of p38/JNK‐associated signaling rather than ERK signaling. Similar decreases in JNK or p38 phosphorylation have been reported for polysaccharides from *Saussurea involucrata* [10] and *Morchella esculenta* [11], respectively.

**FIGURE 7:**
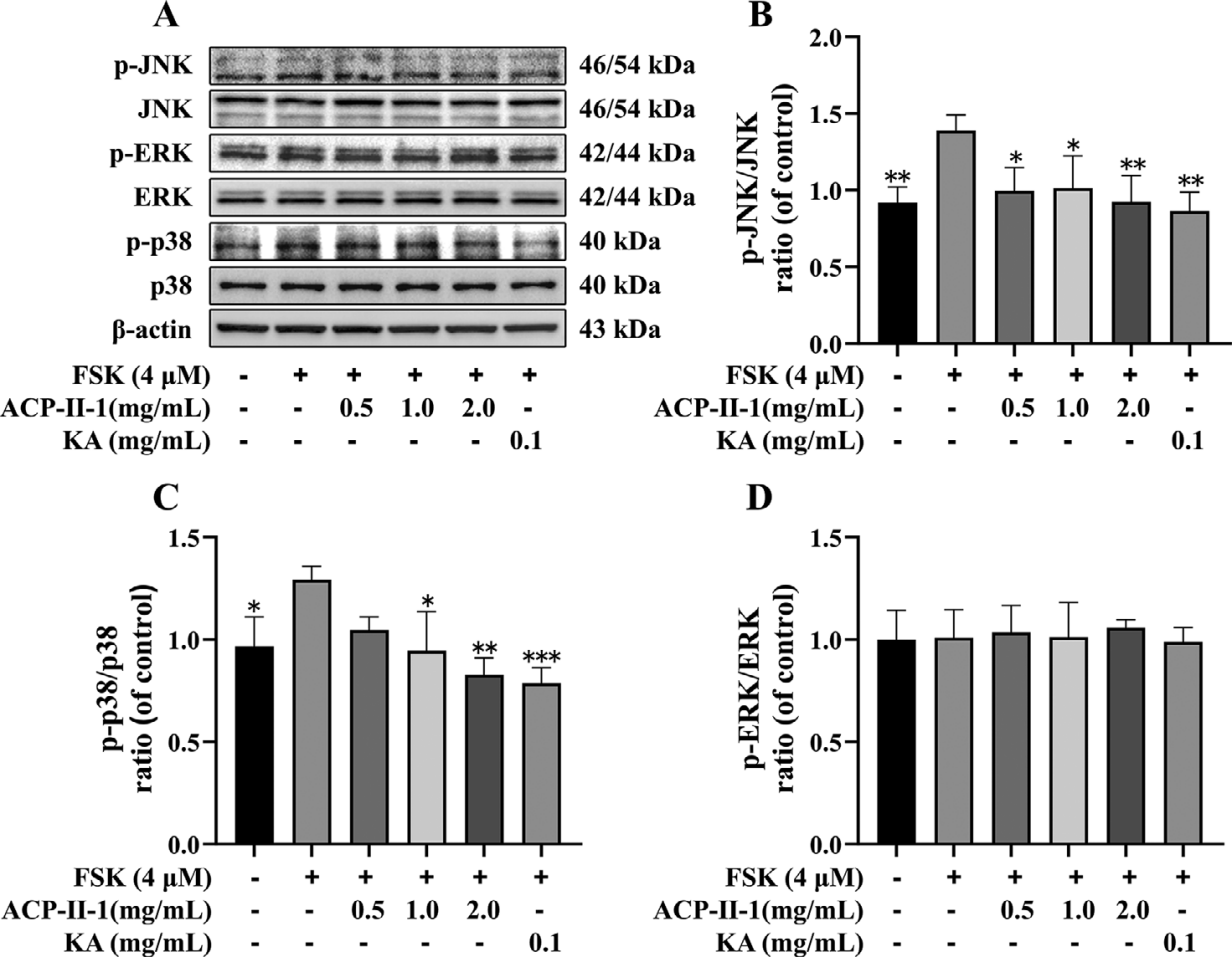
MAPK phosphorylation responses to ACP‐II‐1 in B16F10 cells exposed to FSK. (A) Representative immunoblots of phosphorylated and total JNK, ERK, and p38. (B–D) Densitometric measurements of the p‐JNK/JNK, p‐ERK/ERK, and p‐p38/p38 ratios, expressed relative to the control group. Results are expressed as mean ± SD (*n* = 3). Symbols indicate differences from the FSK group: *p < 0.05, **p < 0.01, and ***p < 0.001.

### 2.10 In Vivo Anti‐Melanogenesis Effect of ACP‐II‐1

The in *vivo* anti‐melanogenic activity of ACP‐II‐1 was subsequently examined in zebrafish larvae. No significant increase in mortality was observed, and no visible morphological abnormalities were detected at 0.5–2.0 mg/mL (Figure S3D), indicating that ACP‐II‐1 was well tolerated under the tested conditions. ACP‐II‐1 treatment increased larval transparency and visibly reduced pigmentation (Figure 8A). Quantitative analyses further showed concentration‐related reductions in melanin content and larval tyrosinase activity (Figure 8B,C). These findings extend the anti‐melanogenic observations in B16F10 cells to an in vivo zebrafish model and agree with previous reports of anti‐melanogenic polysaccharides in zebrafish [11, 54, 55].

**FIGURE 8:**
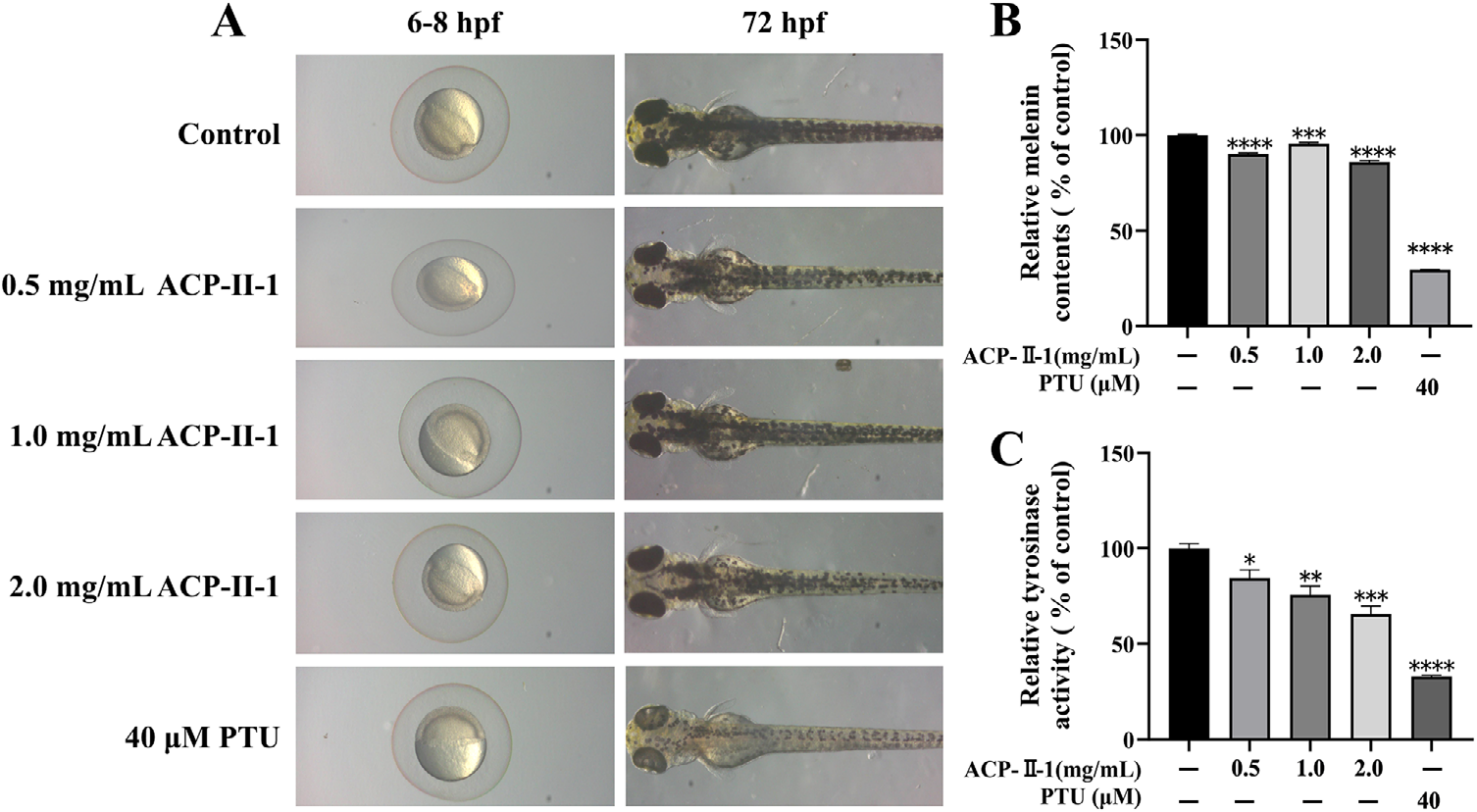
Effect of ACP‐II‐1 on zebrafish pigmentation. Zebrafish embryos were randomly allocated to treatment groups (30 embryos per well; three replicate wells per group) and exposed to the indicated concentrations of ACP‐II‐1, PTU (positive control), or vehicle from 6‐8 to 72 hpf. (A) Representative images of zebrafish larvae at 72 hpf. (B) Relative melanin content and (C) relative tyrosinase activity at 72 hpf. Data in (B) and (C) are presented as mean ± SD (*n* = 3 wells per group), with each well considered the experimental unit. *p < 0.05, **p < 0.01, ***p < 0.001, ****p < 0.0001 versus the vehicle control group.

### 2.11 Integrated Model of ACP‐II‐1‐Associated Anti‐Melanogenic Effects

Based on the above findings, Figure 9 summarizes the molecular and phenotypic changes associated with ACP‐II‐1 treatment. In FSK‐stimulated B16F10 cells, ACP‐II‐1 lowered the ratio of Ser9‐phosphorylated GSK‐3β to total GSK‐3β and reduced β‐catenin abundance. It also diminished the phosphorylation of PKA Cα and CREB and lowered the phosphorylated‐to‐total protein ratios for JNK and p38. These molecular changes coincided with reduced abundance of MITF, TYR, TRP‐1, and TRP‐2, together with declines in cellular tyrosinase activity and melanin accumulation. The model distinguishes experimentally observed changes from literature‐supported regulatory relationships and does not establish direct molecular targeting or causal pathway dependence by ACP‐II‐1.

**FIGURE 9:**
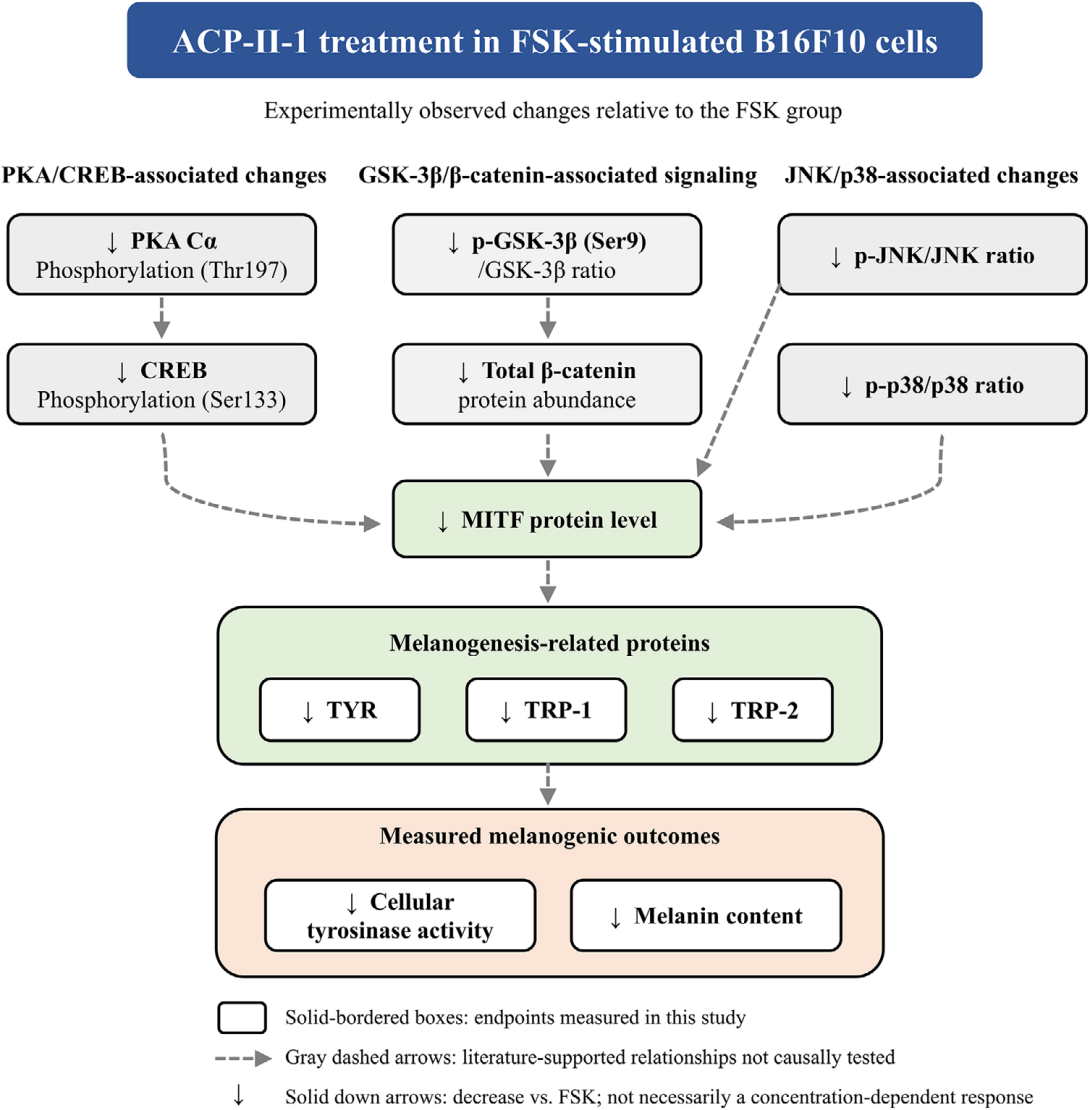
Integrated model of ACP‐II‐1‐associated anti‐melanogenic changes in FSK‐stimulated B16F10 cells. Solid boxes denote endpoints measured in the present study, whereas gray dashed arrows indicate literature‐supported regulatory relationships that were not causally tested. Downward arrows indicate decreases relative to the FSK group and do not necessarily denote concentration‐dependent responses.

### 2.12 Structure–Activity Relationships of ACP‐II‐1

Based on the structural and biological findings, the GalA‐rich pectic architecture and molecular size of ACP‐II‐1 may contribute to its anti‐melanogenic activity. Free GalA has been reported to lower melanin production in α‐MSH‐treated B16 cells and in a three‐dimensional human skin‐equivalent model; it also decreased tyrosinase activity and expression in B16 cells [56]. Likewise, a structurally distinct GalA‐rich pectin from wampee fruit diminished melanin accumulation and melanogenesis‐associated protein abundance in A375 cells exposed to α‐MSH [50]. Together, these observations suggest a possible relationship between a high GalA proportion in pectic materials and anti‐melanogenic effects; however, they do not identify GalpA or HG as the responsible structural determinant because the tested materials differ in polymeric structure, molecular weight, esterification, and branching. Molecular size may represent another contributing factor. ACP‐II‐1 has a molecular weight of 26.145 kDa, and molecular size may also modulate its activity. An enzymatically hydrolyzed *Cuscuta chinensis* polysaccharide with a lower molecular weight exerted a greater inhibitory effect on melanogenesis than its unhydrolyzed counterpart [57]. Nevertheless, enzymatic hydrolysis may alter structural attributes other than molecular size. Because the relevant physicochemical properties were not directly measured, the available data do not permit definition of a specific structure–property relationship. Consequently, no individual structural characteristic can presently be regarded as the sole basis of ACP‐II‐1 activity. Studies using size‐fractionated or selectively modified derivatives are required to clarify its structure–activity relationships.

### 2.13 Limitations and Future Perspectives

Several limitations warrant further investigation. Pathway‐intervention and target‐engagement studies are needed to verify the contributions of the implicated signaling pathways and identify the cellular targets of ACP‐II‐1. Future work should also validate these findings in normal human melanocytes and reconstructed pigmented human skin and assess cutaneous delivery and local safety for topical application. Finally, the structure–activity relationships proposed in Section 2.12 remain preliminary because only native ACP‐II‐1 was examined. Comparisons with size‐fractionated and selectively modified derivatives are required to determine the contributions of individual structural features. Comparisons with size‐fractionated and selectively modified derivatives, together with direct measurements of the relevant physicochemical properties, are required to determine the contributions of individual structural features.

## 3 Conclusion

The extraction process for polysaccharides from *A. camelorum* was optimized, after which the pectic polysaccharide ACP‐II‐1 was isolated. Integrated structural analyses characterized ACP‐II‐1 as a GalA‐rich pectic polysaccharide with a molecular mass of 26.145 kDa, consisting mainly of HG‐type sequences and smaller proportions of branched RG‐I‐type motifs. Under the tested conditions, ACP‐II‐1 lowered melanin accumulation and intracellular tyrosinase activity in FSK‐treated B16F10 cells; reductions in pigmentation and tyrosinase activity were likewise observed in zebrafish. In B16F10 cells, these phenotypic responses coincided with lower abundance of MITF and its downstream melanogenic proteins, as well as altered readouts of GSK‐3β/β‐catenin, PKA/CREB, and p38/JNK signaling. Because these pathway‐related changes neither demonstrate causal dependence nor reveal a direct cellular target, mechanistic interpretations remain limited. Overall, ACP‐II‐1 was structurally characterized as a GalA‐rich pectic polysaccharide and exhibited anti‐melanogenic activity in B16F10 cells and zebrafish. Further validation in mammalian and human‐relevant skin models, together with delivery and safety assessments and direct comparisons of size‐fractionated or structurally modified derivatives, is needed to determine its translational potential and clarify the structural determinants of activity.

## 4 Experimental Section

### 4.1 Materials and Reagents

The aerial parts of *A. camelorum* were collected on 18 July 2023 from Yilah Town, Toksun County, Xinjiang, China. Their botanical identity was confirmed by Prof. Chunfang Lu of XTIPC (Xinjiang Technical Institute of Physics and Chemistry, Chinese Academy of Sciences). The corresponding voucher, numbered WY02719, was archived in the XTIPC Herbarium. Purification was carried out using DEAE‐Sepharose Fast Flow (Cytiva, Uppsala, Sweden), Toyopearl HW‐55F (TOSOH, Tokyo, Japan), and dialysis bags supplied by Spectrum Labs (Rancho Dominguez, CA, USA). Dextran standards (6.0, 9.8, 21.9, 46.5, 110.0, 193.0, 353.0, and 692.0 kDa) were obtained from Shodex (Tokyo, Japan). FSK and phenylthiourea (PTU) were sourced from MCE (Monmouth Junction, NJ, USA). KA was supplied by Solarbio (Beijing, China), whereas L‐3,4‐dihydroxyphenylalanine (L‐DOPA) was purchased through Sigma–Aldrich (St. Louis, MO, USA). Beyotime (China) provided the CCK‐8 kit, whereas ExCell Bio (China) supplied the fetal bovine serum (FBS). B16F10 murine melanoma cells were supplied by the Kunming Cell Bank, Chinese Academy of Sciences (RRID: CVCL_0159). Wild‐type AB zebrafish were sourced through the China Zebrafish Resource Center (Wuhan, China). Antibodies recognizing TYR, TRP‐1, and TRP‐2 were provided by Santa Cruz Biotechnology (USA); those for the other targets were supplied by Cell Signaling Technology (USA). Unless noted otherwise, all remaining chemicals were of analytical‐reagent grade.

### 4.2 Extraction of Polysaccharides

#### 4.2.1 Pretreatment of Plant Material

Dried aerial parts of *A. camelorum* were milled and passed through a 40‐mesh sieve. Following the procedure described in [10], with a modification to the pretreatment duration, the powder was defatted three times with petroleum ether (1:10, w/v) at 25°C for 12 h and subsequently refluxed three times with 95% ethanol (1:10, w/v) for 2 h to remove pigments. The pretreated material was air‐dried and stored until extraction.

#### 4.2.2 Optimization of Extraction of Polysaccharides

##### 4.2.2.1 Single‐Factor Design

Single‐factor experiments followed the procedure in [58] and assessed how extraction temperature (60–100°C), extraction time (1–3 h), liquid‐to‐solid ratio (10–50 mL/g), and cycles number (1–3) affected ACP yield. In each experiment, one factor was varied while the remaining conditions were maintained at 80°C, 2 h, 30 mL/g, and two cycles. Three replicates were performed per condition (*n* = 3). The supernatants were concentrated, combined with ethanol at four times the concentrate volume, kept overnight at 4°C, and centrifuged. The recovered precipitates were lyophilized to obtain crude ACP, after which Equation (1) was used to calculate the extraction yield:

(1) $$Yield\left(\%\right)=\frac{Mass\;of\;freeze-dried\;crude\;ACP\;\left(g\right)}{Mass\;of\;raw\;material\;\left(g\right)}\times 100\%$$

##### 4.2.2.2 Box–Behnken Design

The single‐factor findings guided the design of a three‐factor, three‐level Box–Behnken experiment. Following the Box–Behnken design framework described by Liu et al. [58], Design‐Expert (version 13.0.5) generated 17 runs, five of which were center‐point replicates. The factors were extraction temperature (A), time (B), and liquid‐to‐solid ratio (C); their coded levels and the experimental matrix are provided in Tables S1 and S2, respectively. ACP yield (Y) was fitted to the second‐order polynomial specified by Equation (2):

(2) $$Y=\beta_{0}+\sum_{i=1}^{3}\beta_{i}X_{i}+\sum_{i=1}^{3}\beta_{ii}X_{i}^{2}+\sum_{i=1}^{3}\sum_{j=i+1}^{3}\beta_{ij}X_{i}X_{j}$$

Here, *X*_i_ and *X*_j_ represent the coded factors; *β*_0_, *β*_i_, *β*_ii_, and *β*_ij_ correspond to the intercept and the linear, quadratic, and interaction coefficients, respectively. Model adequacy was evaluated through analysis of variance (ANOVA) at *p* < 0.05, and the model‐predicted optimum was validated experimentally in triplicate.

### 4.3 Isolation and Purification of the Polysaccharides

The separation and purification procedure of crude ACP was adapted from [58], with modifications to the chromatographic media and NaCl elution conditions, as detailed below. It was deproteinized using the Sevag method, dialyzed using a membrane with a molecular‐weight cutoff (MWCO) of 3.5 kDa, and lyophilized. The 500 mg of dried material was reconstituted in distilled water (50 mL) and clarified by centrifugation. The complete supernatant was then transferred to a DEAE‐Sepharose Fast Flow column (60.6 mm × 131 mm). Elution proceeded in three steps with 0, 0.15, and 0.30 M NaCl at 1 mL/min. The anthrone–sulfuric acid assay was used to track carbohydrate in the eluate, and fractions spanning each positive peak were pooled. Each NaCl‐eluted pool was dialyzed (MWCO 3.5 kDa) for 48 h against distilled water, then concentrated and freeze‐dried. The material recovered from the 0.15 M NaCl step, ACP‐II (40 mg), was reconstituted in distilled water (2 mL) and clarified by centrifugation before loading onto a Toyopearl HW‐55F size‐exclusion column (25 × 600 mm). Distilled water, delivered at 0.3 mL/min, served as the eluent. Fractions spanning the selected peak were combined, centrifuged, and lyophilized to obtain the fraction designated ACP‐II‐1.

### 4.4 Structure Elucidation of ACP‐II‐1

#### 4.4.1 Molecular Weight Analysis

Apparent homogeneity and the molecular‐weight parameters (*M*w and *M*n) were examined of ACP‐II‐1 by HPGPC using a procedure described in [19]. The chromatographic configuration consisted of a Waters 1515 system and a refractive‐index detector (Waters, Milford, MA, USA) connected to tandem OHpak SB‐804/806‐HQ columns (Shodex, Tokyo, Japan). Before analysis, the ACP‐II‐1 solution (5 mg/mL) underwent 0.22 µm filtration; the injection volume was 30 µL. Isocratic elution used 0.05 M NaCl at 0.5 mL/min, with the analysis temperature maintained at 40°C. Molecular weight was estimated from a dextran calibration curve relating elution volume to log *M*w.

#### 4.4.2 UV–Visible and FT–IR Spectroscopy Analysis

Both measurements were performed using procedures described in [58]. UV–visible scans were acquired over 190–400 nm on a Shimadzu UV‐2550 spectrophotometer (Kyoto, Japan). FT‐IR data covering 4000–500 cm^−1^ were obtained using a Thermo Fisher Nicolet 6700 spectrometer (Waltham, MA, USA).

#### 4.4.3 Monosaccharide Composition Analysis

An HPLC method based on PMP precolumn derivatization, as described in [35], was used to profile the constituent monosaccharides of ACP‐II‐1. ACP‐II‐1 (5 mg) underwent hydrolysis with 1 mL of 2 M trifluoroacetic acid (TFA) for 2 h at 121°C. Residual TFA was eliminated by repeated methanol co‐evaporation under nitrogen, after which the hydrolysate was redissolved in water. Reference solutions were prepared at 0.5 mg/mL for mannose (Man), rhamnose (Rha), xylose (Xyl), arabinose (Ara), glucose (Glc), glucosamine hydrochloride (GlcN), glucuronic acid (GlcA), galactose (Gal), galacturonic acid (GalA), and galactosamine hydrochloride (GalN), and at 1.0 mg/mL for fucose (Fuc). Aliquots (0.2 mL) of the hydrolyzed sample and individual standard solutions were derivatized separately for 1 h at 70°C with 0.5 M PMP in 0.5 M NaOH. After neutralization and chloroform extraction, HPLC analysis used a Thermo Fisher U3000 system (Waltham, MA, USA) with UV detection and a ZORBAX Eclipse XDB‐C18 column (4.6 × 250 mm, 5 µm; Agilent, Santa Clara, CA, USA) maintained at 70°C. Acetonitrile/phosphate buffer (17:83, v/v) served as the mobile phase at 0.8 mL/min. A 10 µL aliquot was injected, with UV monitoring at 250 nm. Individual monosaccharides were assigned from retention‐time agreement with the corresponding PMP‐derivatized standards. Three replicate analyses were conducted.

#### 4.4.4 Methylation Analysis

The methylation procedure was adapted from [19], with modifications to sample preparation and the carboxyl‐reduction, methylation, and hydrolysis conditions. ACP‐II‐1 (5 mg) was dissolved in distilled water (1 mL) and treated with 0.5 mL of carbodiimide (200 mg/mL) for 2 h. NaBD_4_(70 mg/mL, 1 mL) and imidazole (2 M, 1 mL) were then added for a further 3 h to reduce the carboxyl groups. The reaction was stopped with 300 µL of glacial acetic acid, then dialyzed and lyophilized. The lyophilized product (∼1 mg) was methylated in anhydrous DMSO (1 mL) with NaOH (30 mg, 30 min) and subsequently with CH_3_I (250 µL × 2, each for 1 h). The permethylated material was recovered by dichloromethane extraction. Loss of the O─H stretching band in the FT–IR spectrum confirmed completion of methylation. The product was subsequently hydrolyzed with 2 M TFA for 2 h at 121°C, followed by NaBD_4_ reduction and acetylation with acetic anhydride to form PMAAs. PMAAs were analyzed using an Agilent 7890A GC–MS system coupled to an HP‐5 MS capillary column (30 m × 0.25 mm i.d., 0.25 µm film; Agilent, Santa Clara, CA, USA). The oven program comprised a 1 min hold at 50°C, heating to 130°C at 50°C/min, and further heating to 230°C at 3°C/min, with a final 2 min hold. Helium flowed at 1.0 mL/min; 1 µL was injected using a 10:1 split. Electron‐impact spectra were acquired at 70 eV over *m/z* 30–600. PMAAs were assigned from agreement of their retention times and fragmentation patterns with standard PMAAs and entries in the CCRC Spectral Database. Three replicate analyses were conducted.

#### 4.4.5 SEM–EDX Analysis

Following [58], Surface morphology was imaged using a SUPRA 55VP SEM operated at 20 kV (Zeiss, Oberkochen, Germany), and Elemental data were acquired by energy‐dispersive x‐ray spectroscopy (EDX; Bruker Nano, Berlin, Germany). Samples were platinum‐sputtered prior to analysis to enhance surface conductivity.

#### 4.4.6 XRD Analysis

Diffraction patterns for ACP‐II‐1 were recorded at 25°C on a D8 Advance diffractometer (Bruker, Billerica, MA, USA). Data were collected over 2θ = 5–80° using a 0.01° step size and a dwell time of 0.1 s per step [59].

#### 4.4.7 Congo Red Analysis

The Congo red method [60] was applied to assess whether ACP‐II‐1 adopted a triple‐helical conformation. Solutions containing ACP‐II‐1 (1.0 mg/mL) and Congo red (80 µM) were adjusted to final NaOH concentrations of 0–0.5 M. Following a 10‐min incubation at room temperature, spectra were scanned over 400–700 nm on a UV‐2550 spectrophotometer (Shimadzu, Kyoto, Japan), and changes in λ_max_ were recorded.

#### 4.4.8 NMR Analysis

ACP‐II‐1 (50 mg) in D_2_O (0.5 mL) was examined at 25°C using a VNMRS 600 MHz spectrometer (Varian, Palo Alto, CA, USA). The acquired datasets comprised ^1^H NMR, ^13^C NMR, COSY, HSQC, DEPT‐135, HMBC, and TOCSY spectra.

### 4.5 Anti‐Melanogenesis Activity in B16F10 Cells

#### 4.5.1 Cell Culture and Viability Assay

Cell culture and viability measurements were performed according to [10]. RPMI‐1640 containing 10% FBS and 1% penicillin–streptomycin was used to grow B16F10 cells at 37°C under humidified 5% CO_2_. For viability assessment, cells were plated at 1 × 10^4^ cells/well in 96‐well plates and incubated overnight. Cells were exposed to ACP‐II‐1 (0.05–4.0 mg/mL) for 30 min; FSK (4 µM) was added, and incubation continued for 48 h. After the treatment medium was discarded, each well next received 100 µL of serum‐free medium in which CCK‐8 constituted 10% (v/v). After 1 h at 37°C, absorbance was read at 450 nm, and Equation (3) was used to determine cell viability:

(3) $$Cell\;viability\;\left(\%\right)=\frac{OD_{sample}-OD_{blank}}{OD_{control}-OD_{blank}}\times 100\%$$

#### 4.5.2 Melanin Content Measurement

Melanin measurement was performed according to [10]. B16F10 cells were plated overnight in 6‐well plates (5 × 10^4^ cells/well) and preincubated for 0.5 h with ACP‐II‐1 (0.5, 1.0, or 2.0 mg/mL), KA (0.1 mg/mL; positive control), or fresh medium. FSK (4 µM) was then added to the FSK‐model, KA, and ACP‐II‐1 groups, whereas the untreated control received no FSK; incubation continued for 72 h. Cells were subsequently washed with PBS, lysed in RIPA buffer on ice, and centrifuged for 20 min (12,000 rpm, 4°C) to recover insoluble melanin. Protein in the supernatant was quantified by the BCA assay. The melanin pellet was solubilized in 200 µL of NaOH (1 M) containing 10% DMSO at 80°C for 1 h, and absorbance was read at 405 nm. Melanin values were normalized to total protein and reported relative to the FSK‐model group.

#### 4.5.3 Intracellular TYR Activity Assay

The intracellular TYR assay was performed according to [10]. B16F10 cells were plated overnight in 6‐well plates (5 × 10^4^ cells/well) and preincubated for 0.5 h with ACP‐II‐1 (0.5, 1.0, or 2.0 mg/mL), KA (0.1 mg/mL), or medium alone. FSK (4 µM) was added to the FSK‐model, KA, and ACP‐II‐1 groups but not to the untreated control, and incubation proceeded for 48 h. Cells were washed with ice‐cold PBS and lysed on ice for 20 min in a PBS‐based mixture containing sodium deoxycholate (0.1%) and Triton X‐100 (1%). Following centrifugation for 20 min (12,000 rpm, 4°C), a 90 µL aliquot of the supernatant was combined with 10 µL of 10 mM L‐DOPA in a 96‐well plate. The reaction proceeded at 37°C for 30 min before absorbance was recorded at 490 nm. TYR activity was adjusted for the total protein concentration determined by the BCA assay and expressed relative to the FSK‐model group.

#### 4.5.4 Western Blot Analysis

Immunoblotting was performed using a protocol adapted from [10], with the cell‐collection time adjusted according to the target proteins analyzed. B16F10 cells were plated overnight in 6‐well plates and preincubated for 0.5 h with ACP‐II‐1 (0.5, 1.0, or 2.0 mg/mL), KA (0.1 mg/mL), or medium alone. FSK (4 µM) was added to the FSK‐model, KA, and ACP‐II‐1 groups but not to the untreated control. Cells were collected after 48 h for measurement of TYR, TRP‐1, TRP‐2, and MITF, or after 12 h for measurement of p‐GSK‐3β (Ser9), β‐catenin, GSK‐3β, PKA Cα, p‐PKA Cα (Thr197), p‐CREB (Ser133), p‐JNK, JNK, p‐p38, p38, p‐ERK, and ERK. Cells were lysed in RIPA buffer on ice for 30 min, and the lysates were clarified by centrifugation for 20 min (12,000 rpm, 4°C). Protein concentrations in the supernatants were measured by the BCA assay. Samples containing 30 µg of protein per lane were separated by 10% SDS–PAGE and transferred to PVDF membranes. The membranes were blocked in 5% skimmed milk/TBST for 2 h at room temperature. Membranes were probed with primary antibodies (1:1000) overnight at 4°C. After washing, HRP‐conjugated secondary antibodies (1:2000) were applied for 2 h at room temperature. Bands were detected by ECL and imaged on a ChemiDoc XRS system and quantified with Image Lab v2.0.1 (Bio‐Rad).

### 4.6 Determination of Depigmenting Activity in Zebrafish

#### 4.6.1 Zebrafish Husbandry and Embryotoxicity Assay

Adult wild‐type AB zebrafish (Danio rerio) were kept in a recirculating system at 26°C ± 2°C under a 14‐h light/10‐h dark schedule and were fed three times daily. Naturally spawned embryos were collected within 1.5 h post‐fertilization (hpf), washed, and reared in sterile embryo medium. The embryotoxicity assay followed the protocols in [11], with modifications to the embryo‐exposure conditions described below. Healthy embryos (6‐8 hpf) were randomized to 48‐well plates (5 embryos/well; 3 parallel wells/treatment), and each well received 1 mL of an ACP‐II‐1 solution (0.125–4.0 mg/mL). Embryos were incubated to 72 hpf and examined daily under a stereomicroscope. At 72 hpf, 4.0 mg/mL was identified as the highest tested safe concentration, with both mortality and malformation rates below 20%. For consistency with the cell assays, 0.5, 1.0, and 2.0 mg/mL were selected for subsequent zebrafish assays. Ethical approval was obtained from the Experimental Animal Ethics Committee of Guangdong Pharmaceutical University (approval no. gdpulac2025361).

#### 4.6.2 Assessment of Depigmentation and TYR Activity in Zebrafish

The assessment of depigmentation and TYR activity was adapted from [11], with modifications to the exposure and assay conditions described below. Healthy embryos (6‐8 hpf) were exposed in 6‐well plates (30 embryos/well; 3 parallel wells/group) to ACP‐II‐1 (0.5, 1.0, or 2.0 mg/mL), PTU (40 µM; positive control), or vehicle until 72 hpf, when pigmentation was photographed. Larvae were sonicated in 500 µL of sodium deoxycholate buffer (5 mg/mL), and the homogenate was centrifuged for 20 min (12,000 rpm, 4°C). For melanin measurement, the pellet was solubilized in 150 µL of 1 M NaOH at 80°C for 1 h, and absorbance was read at 405 nm. For TYR activity, 100 µL of the supernatant was combined with 100 µL of 5 mM L‐DOPA and incubated at 37°C for 1 h, and read at 475 nm. Both endpoints were adjusted for total protein quantified by the BCA assay and expressed relative to the vehicle‐control group.

### 4.7 Statistical Analysis

Data are presented as mean ± SD. Cell‐based results were obtained from three independent experiments, whereas zebrafish results were based on three replicate wells per group, with each well considered the experimental unit. Normality was examined using the Shapiro–Wilk test and variance homogeneity using Levene's test. Group differences were evaluated by one‐factor ANOVA with Tukey‐adjusted multiple comparisons in GraphPad Prism 10.1.2. The significance threshold was *p* < 0.05. Origin 2021 and Adobe Photoshop CS6 were used for figure preparation.

## Author Contributions

**Zhitao Liu**: writing – original draft, methodology, validation, visualization, investigation, data curation. **Shuyan Han**: writing– review and editing, methodology, investigation, validation. **Nannan Xu**: writing – review and editing, project administration, funding acquisition. **Nuermaimaiti Abudukelimu**: resources, supervision, investigation, project administration, formal analysis. **Jiangyu Zhao**: writing– review and editing, supervision, project administration, funding acquisition, conceptualization.

## Conflicts of Interest

The authors declare no conflicts of interest.

## Supporting information




**Supporting File 1**: cbdv71878‐sup‐0001‐SuppMat.pdf

## Data Availability

The data that support the findings of this study are available from the corresponding author upon reasonable request.
