# Supplementary material for: Isolation, Structural Elucidation, and Anti‐Melanogenic Activity of a Novel Pectic Polysaccharide From Alhagi camelorum Fisch
Source: Chem Biodivers. 2026 Oct 30;23(11):e71878. doi: 10.1002/cbdv.71878 (PMC13720961; doi:10.1002/cbdv.71878)
Supplement: Supplementary file 1 [file CBDV-23-e71878-s001.pdf]

Supplementary Material-Figure

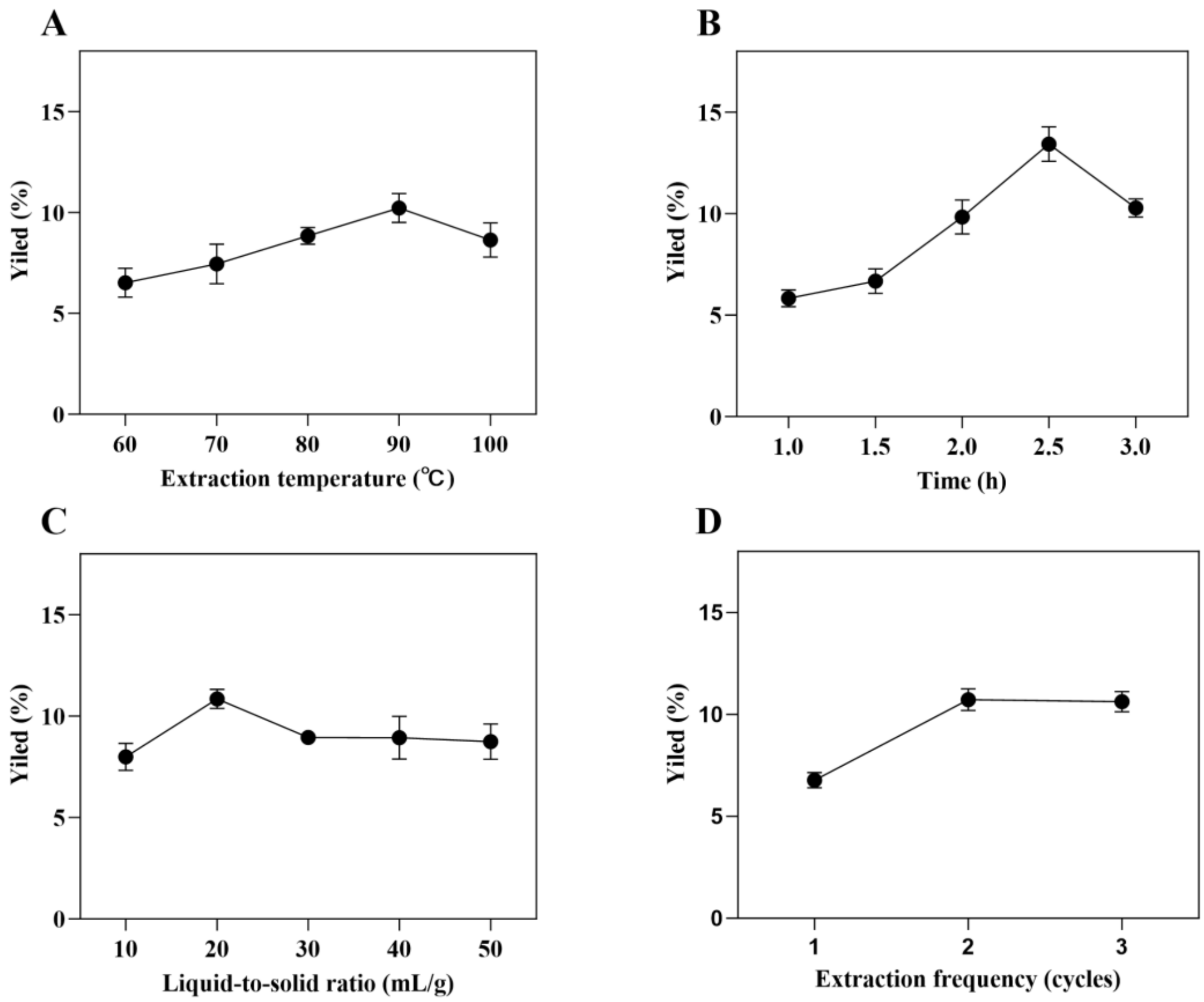

Figure S1. Effects of extraction temperature (A), time (B), liquid-to-solid ratio (C), and cycle number (D) on the yield of ACP in single-factor tests.

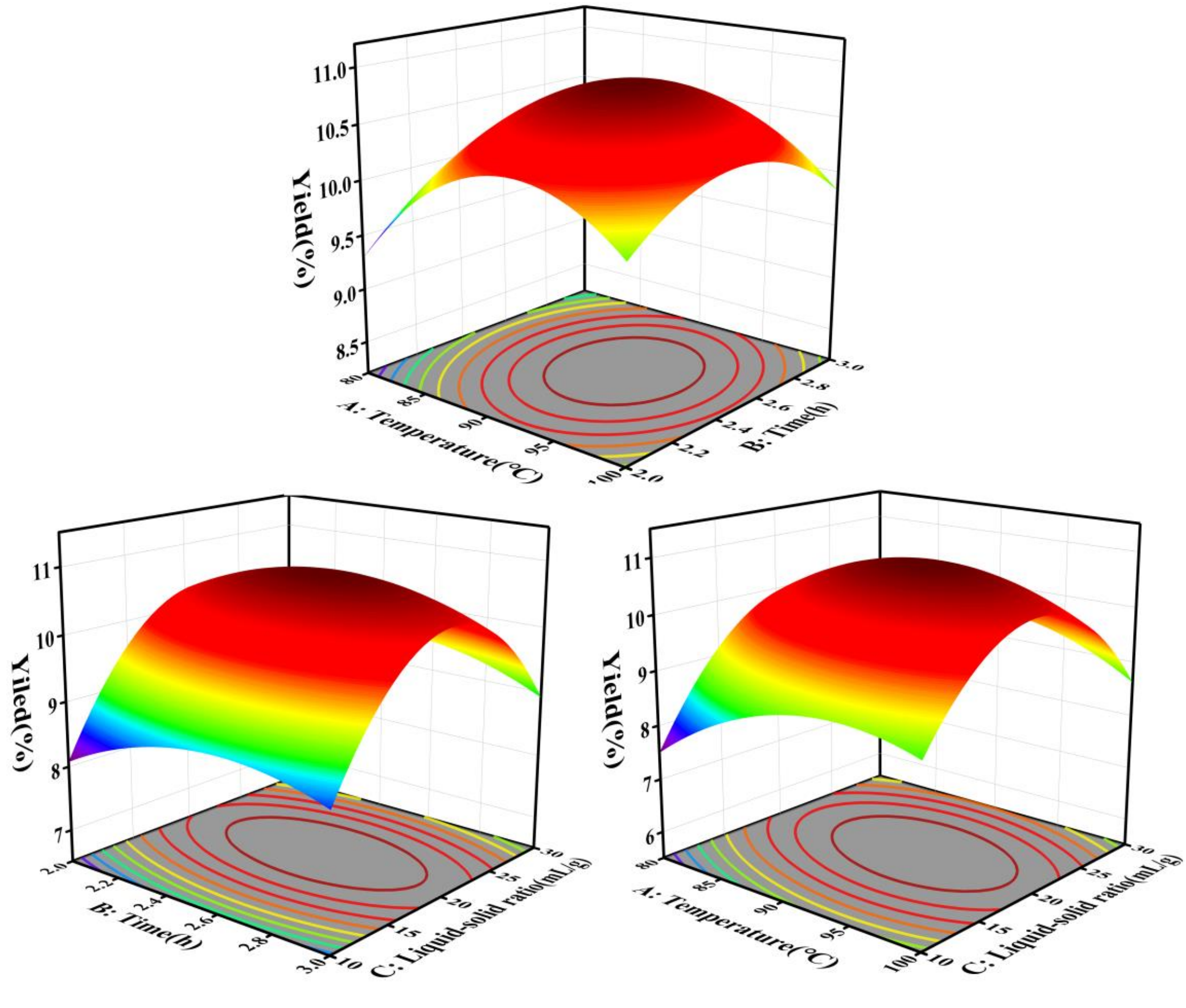

Figure S2. Three-dimensional response surface plots depicting the interactive effects of variable pairs on the yield of ACP.

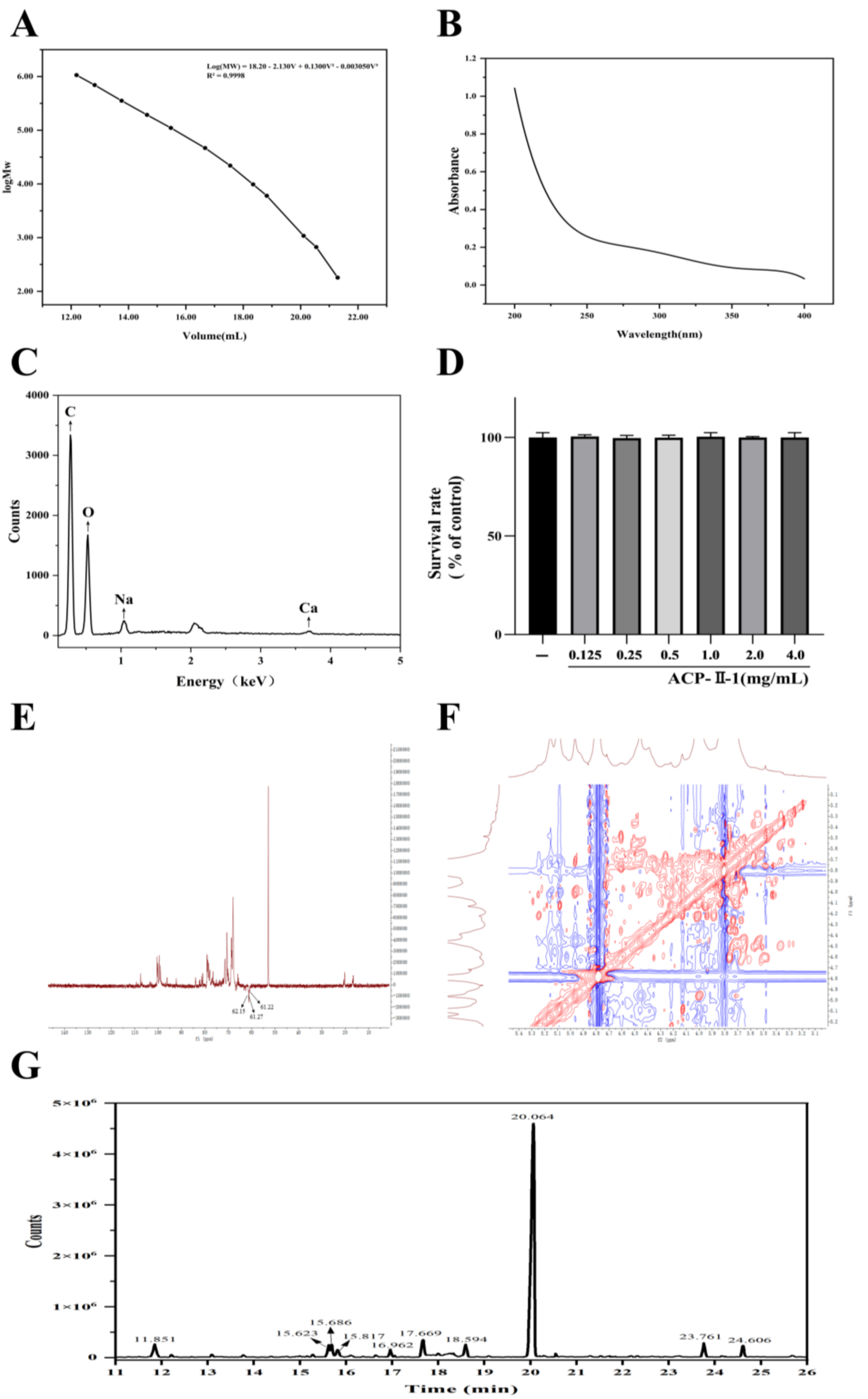

Figure S3. Comprehensive analysis of ACP-II-1. (A) Molecular weight calibration curve. (B) UV-Vis spectrum. (C) EDX analysis. (D) Zebrafish toxicity assay. (E) DEPT-135 NMR spectrum. (F) TOCSY NMR spectrum. (G) PMAA-GC-MS chromatogram (TIC).

Supplementary Material-Table

Table S1. Coded and actual levels of independent variables used in the Box–Behnken design.

Independent variables	Symbol	Coded levels		
		-1	0	1
Temperature (°C)	A	80	90	100
Time (h)	B	2	2.5	3
Liquid-solid ratio (mL/g)	C	10	20	30

Table S2 Box-Behnken design and response values of the crude ACP yields.

RUN	Temperature (°C)	Time (h)	Liquid-to-solid ratio（mL/g）	yield（%）
1	90	3	10	8.625
2	90	2.5	20	11.5
3	80	2.5	30	9
4	90	2.5	20	11.25
5	100	2.5	10	8.625
6	90	2.5	20	10.25
7	100	3	20	9.75
8	100	2.5	30	8.625
9	80	2	20	9.375
10	90	2	30	9
11	80	3	20	9.5
12	90	3	30	9
13	80	2.5	10	7.5
14	90	2.5	20	10.5
15	90	2	10	8
16	90	2.5	20	10.75
17	100	2	20	10

Table S3 ANOVA for the quadratic regression model of ACP yield.

Source	Sum of Squares	df	Mean Square	F-Value	*P*-value	
**Model**	18.66	9	2.07	12.63	0.0015	significant
A-Temperature	0.3301	1	0.3301	2.01	0.1992	
B-Time	0.0313	1	0.0313	0.1903	0.6758	
C-Liquid-solid ratio	1.03	1	1.03	6.29	0.0405	
AB	0.0352	1	0.0352	0.2141	0.6576	
AC	0.5625	1	0.5625	3.43	0.1066	
BC	0.0977	1	0.0977	0.5948	0.4658	
A^2	2.10	1	2.10	12.79	0.0090	
B^2	1.00	1	1.00	6.10	0.0429	
C^2	12.26	1	12.26	74.66	<0.0001	
Residual	1.15	7	0.1642			
Lack of Fit	0.0742	3	0.0247	0.0921	0.9606	not significant
Pure Error	1.07	4	0.2687			
Cor Total	19.81	16				
**Model statistics**						
R^2					0.9420	
Adjusted R^2					0.8674	
Predicted R^2					0.8553	
C.V. (%)					4.27	
Adequate precision					10.7800	

Table S4. Yields and preliminary anti-melanogenic activities of polysaccharide fractions (ACP-I, ACP-II, and ACP-III) from *Alhagi camelorum* Fisch.

Fraction	Yield (%)	Melanin content inhibition rate (%) at 1.0 mg/mL
**ACP-II**	**38.77**	**28.72 ± 2.37**
ACP-I	11.08	-5.37 ± 1.49
ACP-III	3.54	9.07 ± 1.21

Note:

1. Yield was calculated as (weight of freeze-dried fraction / weight of deproteinized and dialyzed *A. camelorum* polysaccharide (ACP) loaded onto the DEAE-Sepharose column) × 100%.

2. B16F10 melanoma cells were treated with 1.0 mg/mL of each fraction for 72 h after forskolin (FSK, 4 μM) stimulation. Melanin content was measured and normalized to total protein content. Data are presented as mean ± SD (n = 3). The inhibition rate was calculated relative to the FSK-induced model group (set as 0% inhibition). Negative values indicate a stimulatory effect on melanin synthesis.

3. **Bold** indicates the fraction selected for further purification based on the highest yield and the strongest melanin inhibitory effect.
